# Supplementary material for: Optimizing vaccine allocation for COVID-19 vaccines shows the potential role of single-dose vaccination
Source: Nat Commun. 2021 Jun 8;12:3449. doi: 10.1038/s41467-021-23761-1 (PMC8187351; doi:10.1038/s41467-021-23761-1)
Supplement: Supplementary file 1 — Suplementary information [file 41467_2021_23761_MOESM1_ESM.pdf]

Supplemental Information for "Optimizing vaccine allocation for COVID-19 vaccines shows the potential role of single-dose vaccination."

Laura Matrajt,[1*] Julia Eaton,[2] Tiffany Leung,[1] Dobromir Dimitrov,[1,3]
Joshua T. Schiffer,[1,4,5] David A. Swan,[1] Holly Janes[1]

[1]Vaccine and Infectious Disease Division, Fred Hutchinson Cancer Research Center, Seattle, WA, USA
[2] School of Interdisciplinary Arts and Sciences, University of Washington, Tacoma, USA
[3] Department of Applied Mathematics, University of Washington, Seattle, WA, USA
[4] Department of Medicine, University of Washington, Seattle, WA, USA
[5]Clinical Research Division, Fred Hutchinson Cancer Research Center, WA, USA
[*]To whom correspondence should be addressed; E-mail: laurama@fredhutch.org

Supplemental Figures

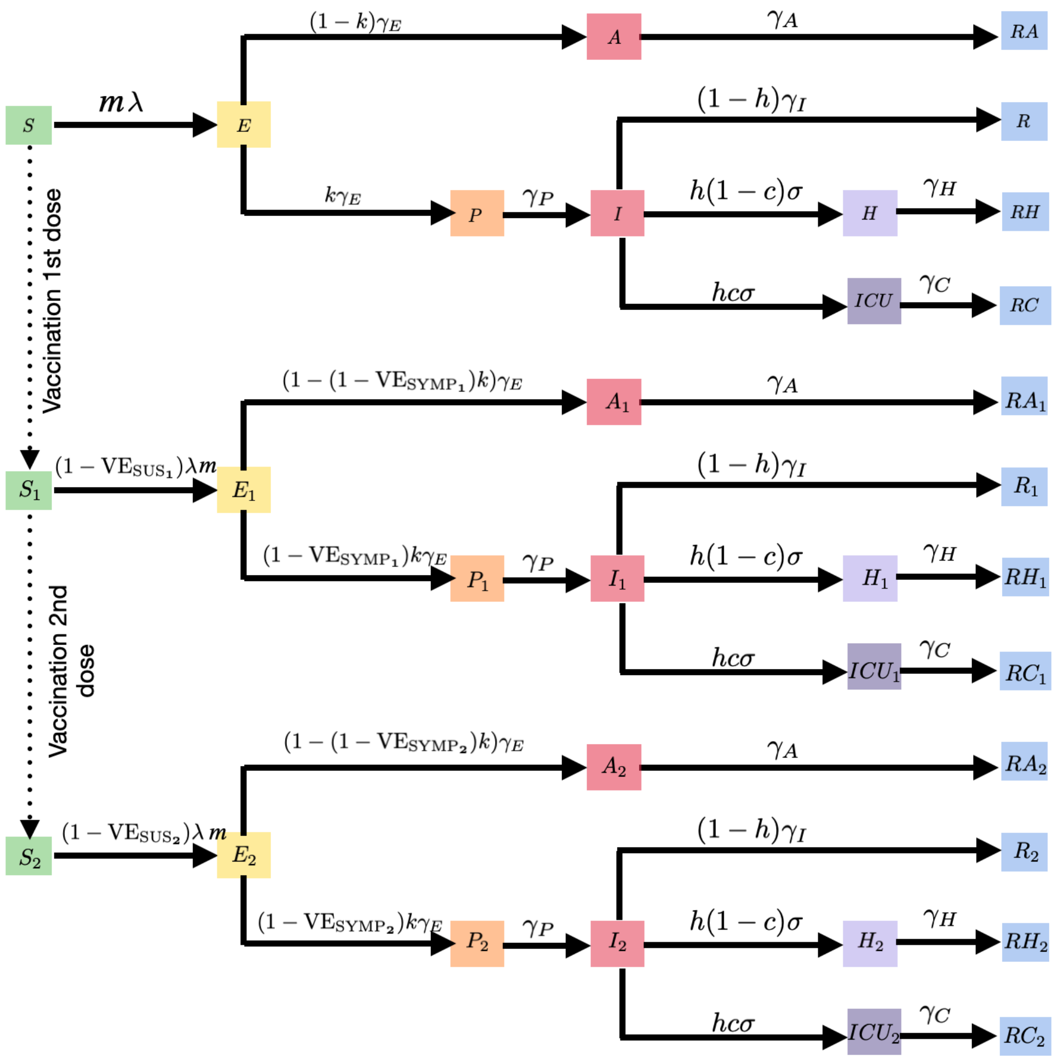

Supplementary Figure 1: Diagram of the SEIR model with vaccination with one or two doses of vaccine. Age indices have been omitted for clarity. For each age group, our model tracks susceptible S, exposed E, asymptomatic A, pre-symptomatic P and symptomatic infected individuals classed by disease severity. Symptomatic individuals have one of three fates: they become mildly symptomatic I, hospitalized in a non-ICU ward H, or hospitalized requiring intensive care, ICU. After infection, individuals move to the respective recovered classes: recovered asymptomatic RA, recovered mildly symptomatic R, recovered non-ICU hospitalized RH, and ICU hospitalized recovered RC. Individuals who received one or two doses of vaccine go through analagous compartments indexed by $j = 1, 2$ for one and two doses respectively. Age indices have been omitted for clarity. Refer to Supplementary Table 1 and Supplementary Table 2 for the definitions of all parameters.

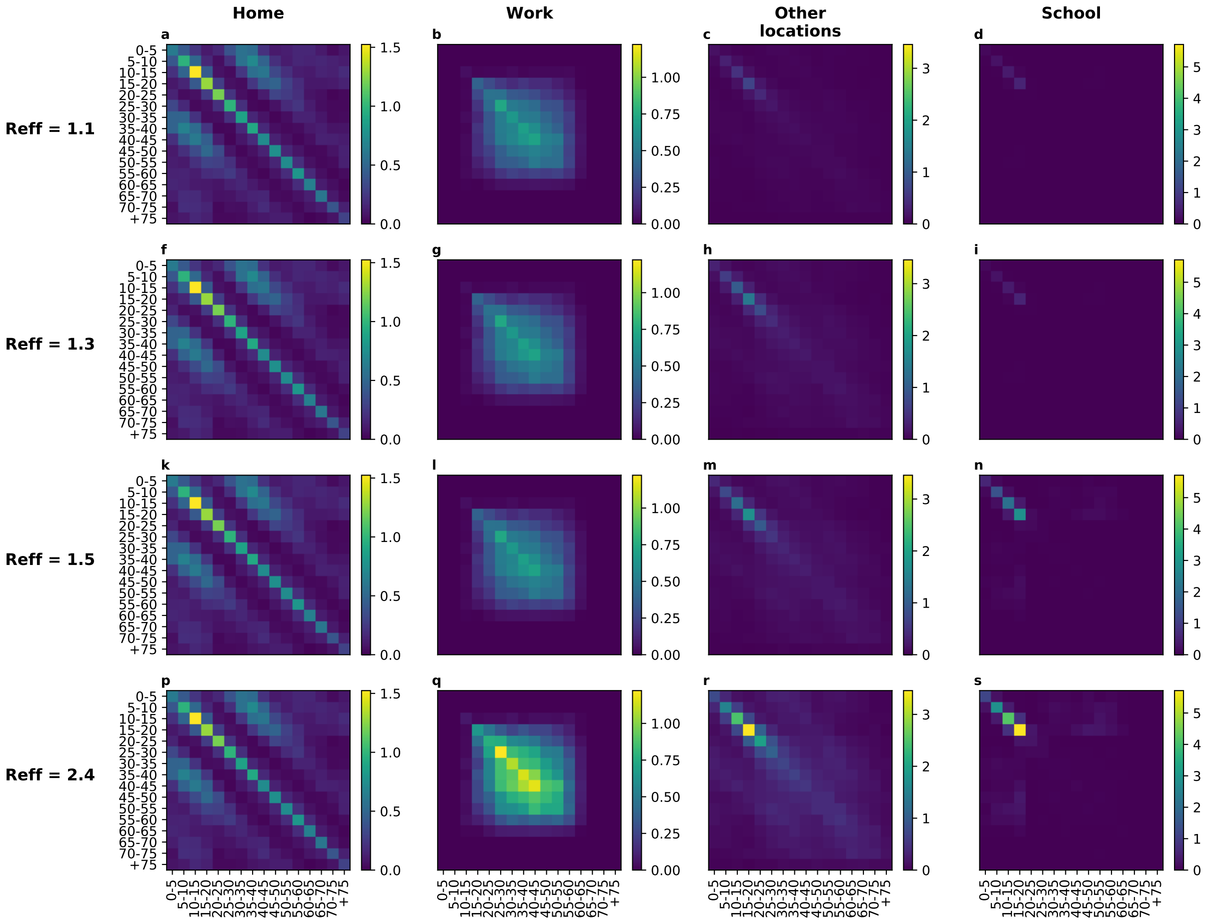

Supplementary Figure 2: Modified contact matrices considered in our simulations. The contact matrices given in [1] were modified with the multipliers given in table 3 for each of the four scenarios of viral transmission considered in our simulations, resulting in an effective reproductive number R_{eff} = 1.1 (a–d), 1.3 (f–i), 1.5 (k–n), and 2.4 (p–s).

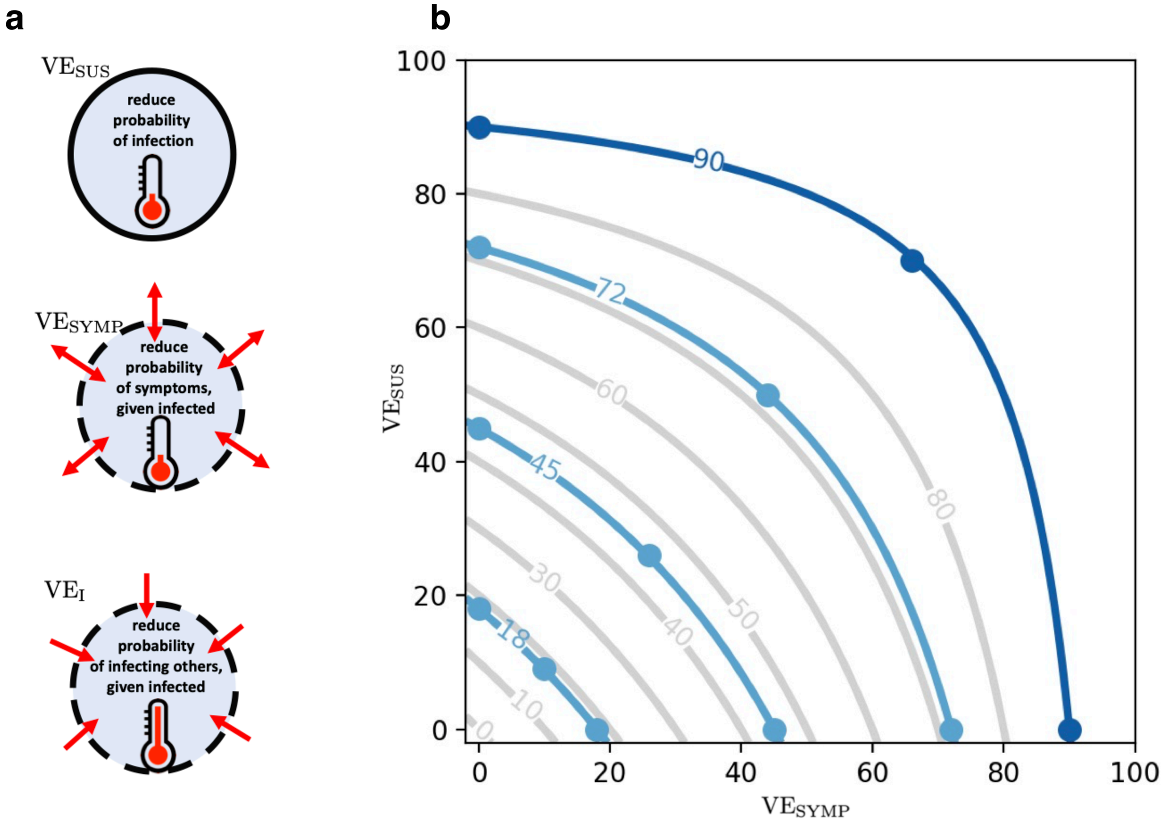

Supplementary Figure 3: a. Different vaccine effects modeled. A vaccine can reduce the probability of infection, denoted by $\mathrm{VE_{SUS}}$. In addition, it can reduce the probability of developing symptoms once infected, denoted $\mathrm{VE_{SYMP}}$. Finally, it can reduce the infectiousness of a vaccinated person upon infection, denoted $\mathrm{VE_{I}}$. We assumed that the vaccine efficacy against disease $\mathrm{VE_{DIS}}$ can be expressed as a combination of $\mathrm{VE_{SUS}}$ and $\mathrm{VE_{SYMP}}$ (see text). b. Level curves for $\mathrm{VE_{DIS}}$ as a function of $\mathrm{VE_{SUS}}$ and $\mathrm{VE_{SYMP}}$. The light blue lines indicate the vaccine efficacies $\mathrm{VE_{DIS_1}}$ obtained after a first dose of vaccine considered in the main analysis. The dark blue line indicates the vaccine efficacy obtained after the full dosage (two doses) $\mathrm{VE_{DIS}} = 90\%$.

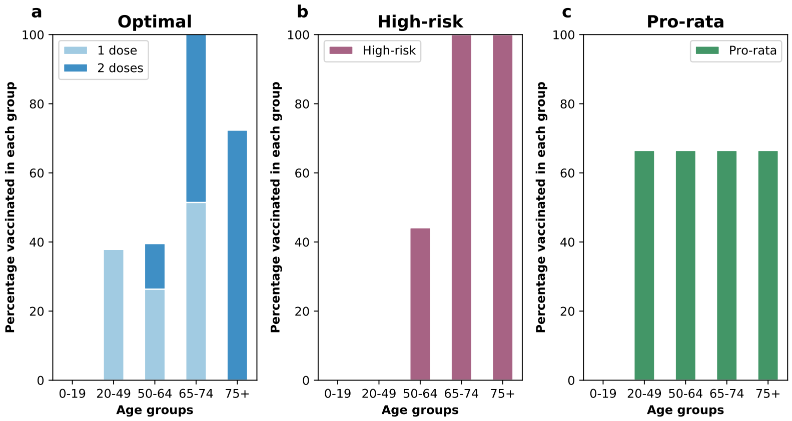

Supplementary Figure 4: Example of the A. optimal, B. high-risk and C. pro-rata strategies. Here, we assumed enough vaccine to cover 50% of the population. The optimal strategy allocates vaccine as determined by our optimization routine. The high-risk strategy allocates two doses of vaccine starting with the oldest age group and then to other age groups in decreasing order. The pro-rata strategy allocates one-dose of vaccine to all the adult groups in the population proportional to their size.

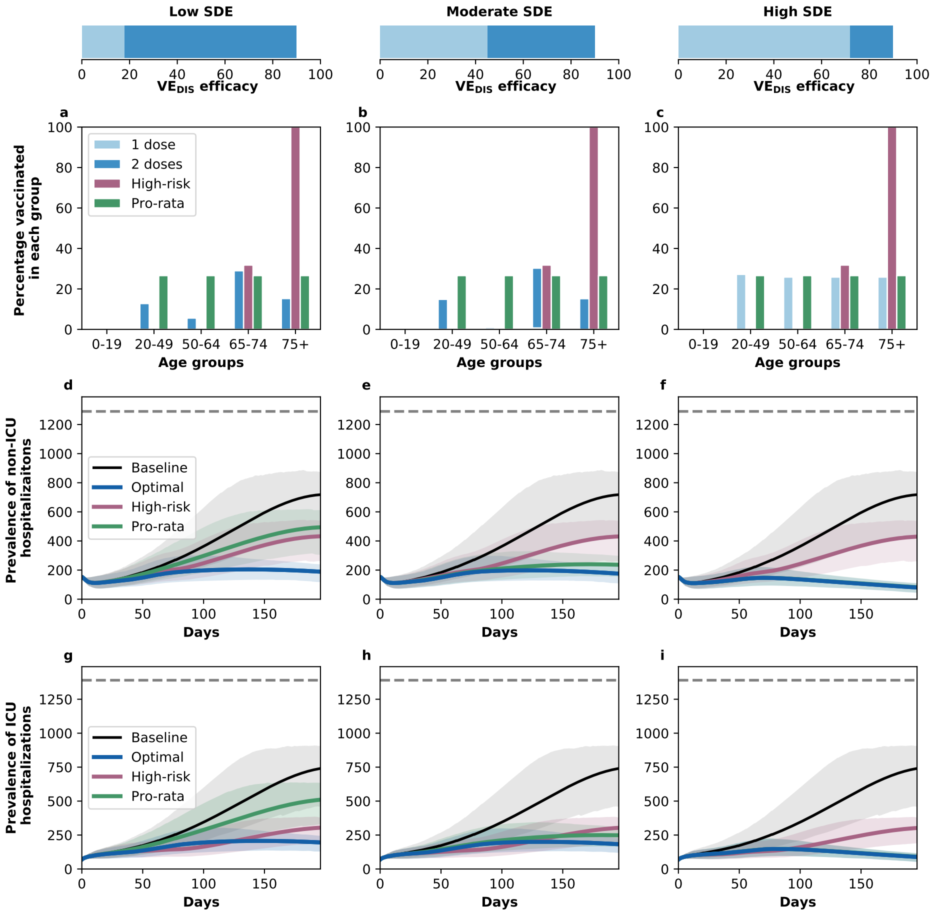

Supplementary Figure 5: a–c: Optimal, pro-rata and high-risk strategies to minimize deaths with enough vaccine to cover 20% of the population with a single dose (10% with two doses). Optimal (light and dark blue), high-risk (pink) and pro-rata (green) allocation strategies. Within each panel, the bars represent the percentage vaccinated in each vaccination group. d–f: Prevalence of non-ICU hospitalizations. Prevalence of non-ICU hospitalizations in absence of vaccine (black), with the optimal allocation strategy to minimize deaths (blue), high-risk strategy (pink) or the pro-rata strategy (green), the gray dashed line indicates the 10% occupancy of non-ICU beds in WA state. g–i: Prevalence of ICU hospitalizations. Prevalence of non-ICU hospitalizations in absence of vaccine (black), with the optimal allocation strategy to minimize deaths (blue), the high-risk strategy (pink) or the pro-rata strategy (green), the gray dashed line indicates the total number of ICU beds in WA state. Shaded areas represent central 95% of 1000 simulations (uncertainty intervals, see SM for full details). The columns correspond to assumptions that the single-dose efficacy (SDE) is low (left column, $VE_{DIS_1} = 18\%$), moderate (center column, $VE_{DIS_1} = 45\%$) or high (right column, $VE_{DIS_1} = 72\%$), corresponding 20, 50 or 80% of the 90% efficacy that is assumed following two doses of vaccine, respectively.

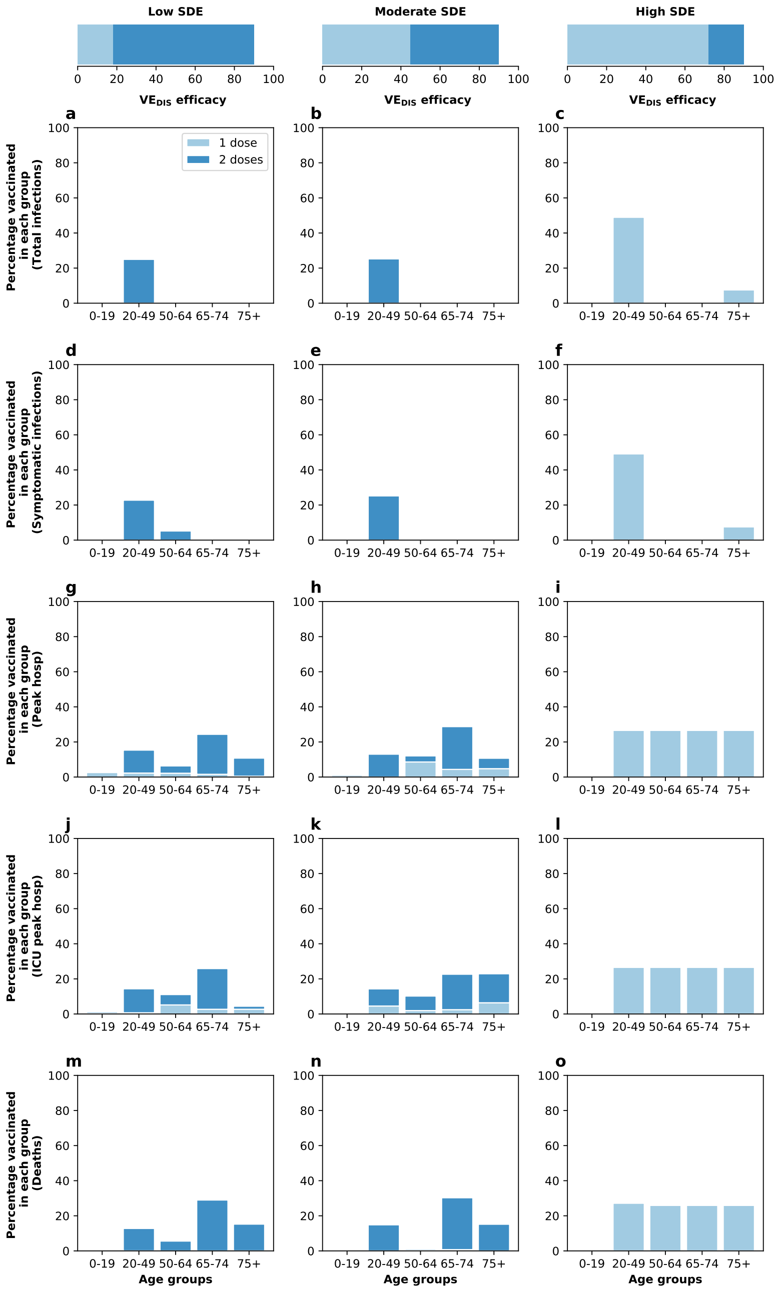

Supplementary Figure 6: Optimal vaccine allocation strategies for different disease metrics with 20% coverage. Optimal vaccine allocation assuming enough vaccine to cover 20% of the population with a single dose (10% with two doses). Each row represents a different disease metric minimized: cumulative infections (a–c), cumulative symptomatic infections (d–f), non-ICU peak hospitalizations (g–i), ICU hospitalizations (j–l) and total deaths (m–o). The columns correspond to assumptions that the single-dose efficacy (SDE) is low (left column, $VE_{DIS_1} = 18\%$), moderate (center column, $VE_{DIS_1} = 45\%$) or high (right column, $VE_{DIS_1} = 72\%$), corresponding 20, 50 or 80% of full vaccine efficacy, $VE_{DIS} = 90\%$, assumed following two doses of vaccine, respectively. Here, we assumed an effective reproductive number $R_{eff} = 1.1$.

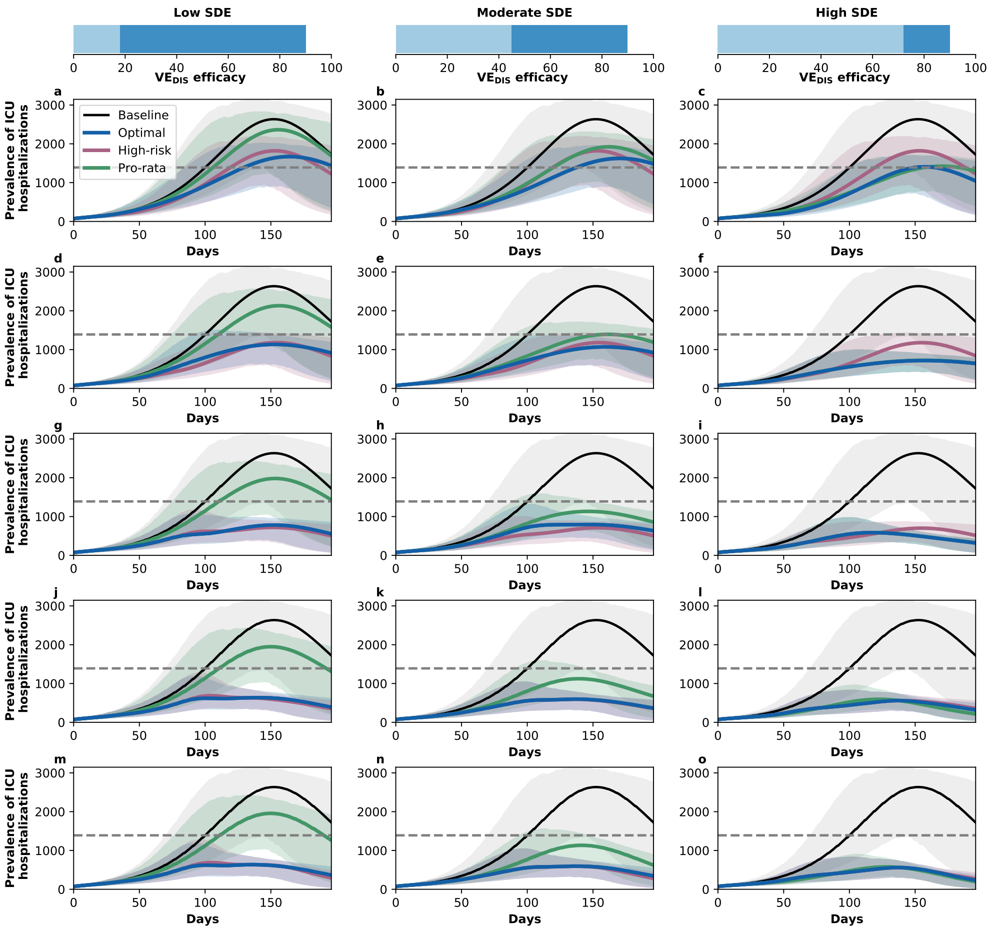

Supplementary Figure 7: Prevalence of ICU hospitalizations with R_{eff} =1.3. Prevalence of ICU hospitalizations in absence of vaccine (black), with the optimal allocation strategy to minimize ICU hospitalizations (blue), the high-risk strategy (pink) or the pro-rata strategy (green). The gray dashed line indicates the total number of ICU beds in WA state. Each row corresponds to a different vaccination coverage, ranging from 10% (a–c) to 50% (m–o) coverage with a single dose. Shaded areas represent central 95% of 1000 simulations (uncertainty intervals, see SM for full details). The columns correspond to assumptions that the single-dose efficacy (SDE) is low (left column, VE_{DIS_1} = 18%), moderate (center column, VE_{DIS_1} = 45%) or high (right column, VE_{DIS_1} = 72%), corresponding 20, 50 or 80% of the full vaccine efficacy, VE_{DIS} = 90% assumed following two doses of vaccine, respectively. Here, we assumed an effective reproductive number R_{eff} = 1.1.

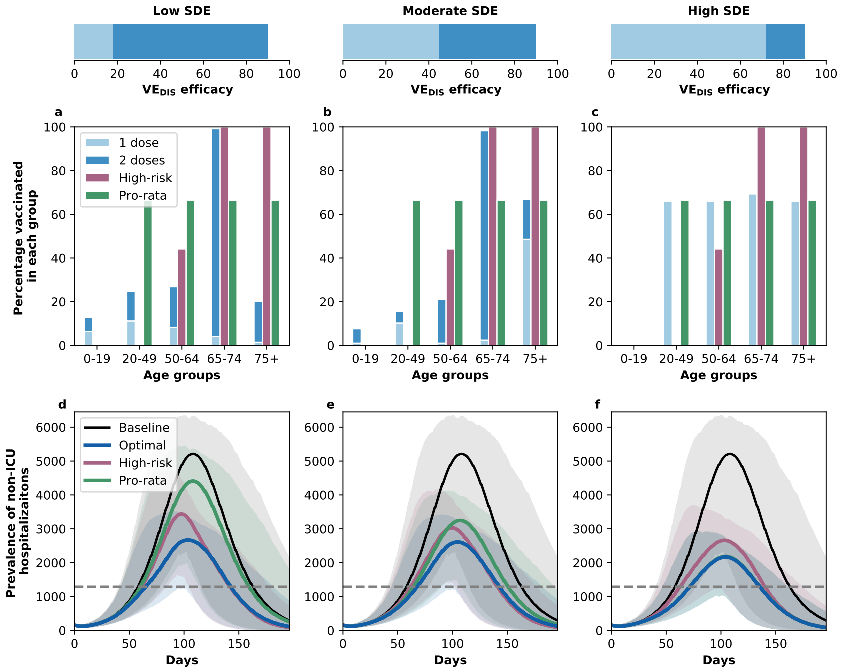

Supplementary Figure 8: a–c: Optimal strategy to minimize non-ICU hospitalizations, pro-rata and high-risk strategies with enough vaccine to cover 50% of the population with a single dose (25% with two doses) with an effective reproductive number R_{eff} =1.5. Optimal allocation strategy to minimize peak non-ICU hospitalizations (light and dark blue), high-risk (pink) and pro-rata (green) strategies. Within each panel, the bars represent the percentage vaccinated in each vaccination group. d–f: Prevalence of non-ICU hospitalizations. Prevalence of non-ICU hospitalizations in absence of vaccine (black), with the optimal allocation strategy to minimize non-ICU hospitalizations (blue), the high-risk strategy (pink) or the pro-rata strategy (green). The gray dashed line indicates 10% occupancy of non-ICU beds in WA state. Shaded areas represent central 95% of 1000 simulations (uncertainty intervals, see SM for full details). The columns correspond to assumptions that the single-dose efficacy (SDE) is low (left column, $VE_{DIS_1} = 18\%$), moderate (center column, $VE_{DIS_1} = 45\%$) or high (right column, $VE_{DIS_1} = 72\%$), corresponding 20, 50 or 80% of the full vaccine efficacy, $VE_{DIS} = 90\%$ assumed following two doses of vaccine, respectively.

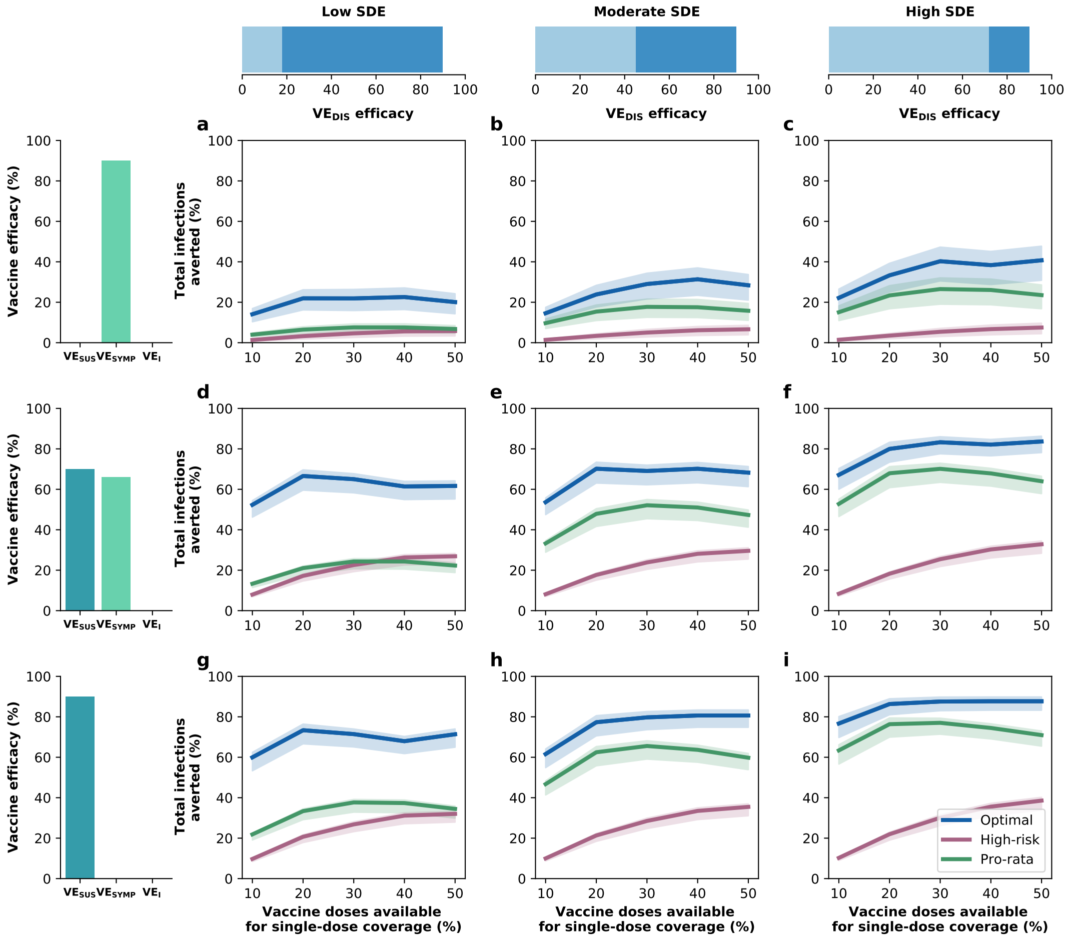

Supplementary Figure 9: Percentage of cumulative infections averted for different vaccine profiles. Percentage of cumulative infections averted for the optimal allocation strategy to minimize infections (blue), the high-risk strategy (pink) and the pro-rata strategy (green) strategies with enough vaccine to cover 10–50% of the population with one dose. Each row represents a different breakdown of vaccine efficacy against disease after two doses $VE_{DIS} = 90\%$ as a function of the vaccine efficacy reducing susceptibility to infection, VE_{SUS}, and the vaccine efficacy reducing the probability of developing COVID-19 symptoms upon infection, VE_{SYMP}. Top row (a–c): VE_{DIS} is exclusively mediated by a reduction in symptoms upon infection. Middle row (d–f): VE_{DIS} is mediated by a combination of reduction in susceptibility to infection and reduction of symptoms upon infection. Bottom row (g–i): VE_{DIS} is exclusively mediated by a reduction in susceptibility to infection. The columns correspond to assumptions that the single-dose efficacy (SDE) is low (left column, $VE_{DIS_1} = 18\%$), moderate (center column, $VE_{DIS_1} = 45\%$) or high (right column, $VE_{DIS_1} = 72\%$), corresponding 20, 50 or 80% of the 90% efficacy that is assumed following two doses of vaccine, respectively. Shaded areas represent central 95% of 1000 simulations (uncertainty intervals, see SM for full details). Here, we assumed an effective reproductive number $R_{eff} = 1.1$.

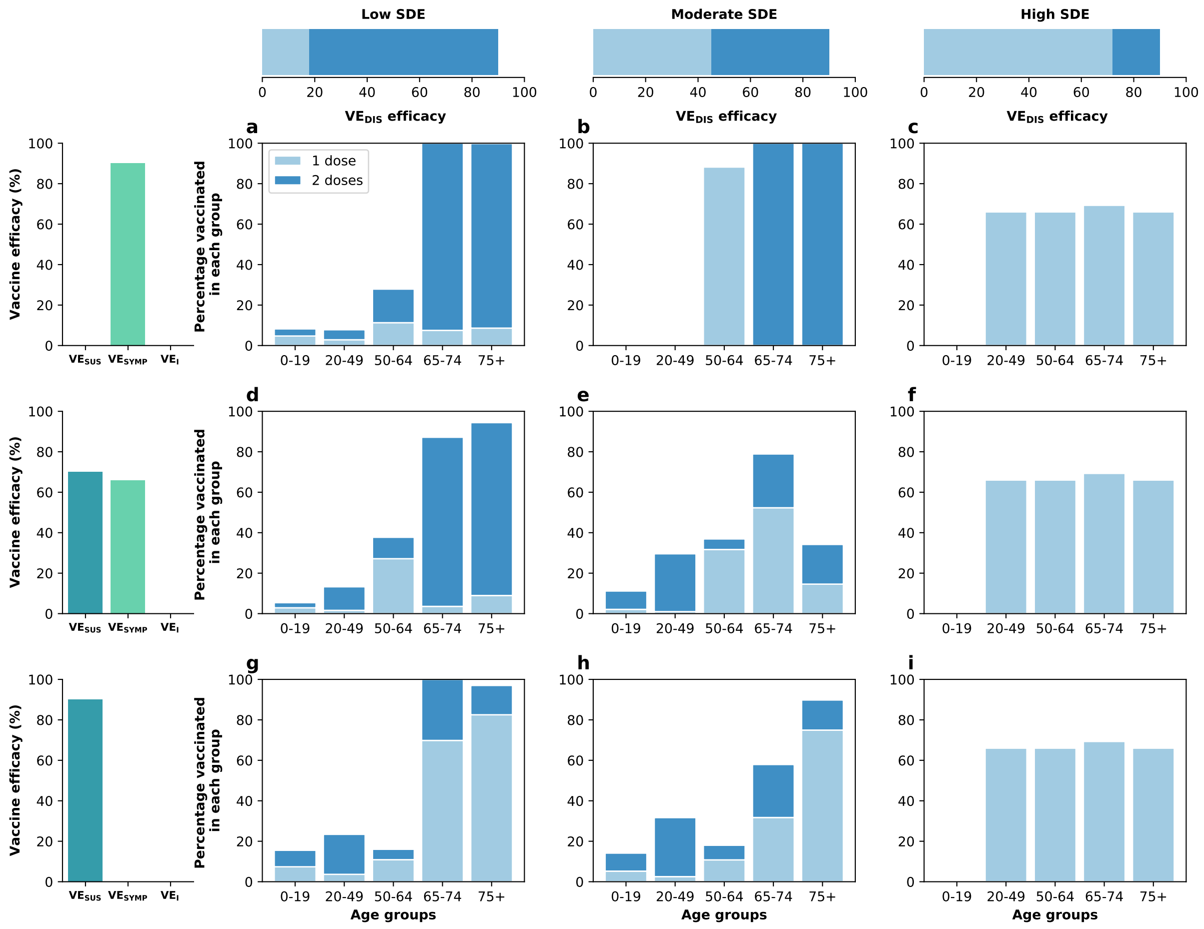

Supplementary Figure 10: Optimal vaccine allocation to minimize deaths for different vaccine profiles with 50% coverage and assuming asymptomatic infections are 30% as infectious as symptomatic infections. Optimal vaccine allocation for minimizing deaths for a assuming enough vaccine to cover 50% of the population with a single dose (25% with two doses). For each panel (A-I), the bars represent the total percentage of the population in each vaccination group to be vaccinated, split in those receiving a single dose (light blue) and those receiving two doses (dark blue). Each row represents a different breakdown of vaccine efficacy against disease after two doses $VE_{DIS} = 90\%$ as a function of the vaccine efficacy reducing susceptibility to infection, VE_{SUS}, and the vaccine efficacy reducing the probability of developing COVID-19 symptoms upon infection, VE_{SYMP}. Top row (a–c): VE_{DIS} is exclusively mediated by a reduction in symptoms upon infection. Middle row (d–f): VE_{DIS} is mediated by a combination of reduction in susceptibility to infection and reduction of symptoms upon infection. Bottom row (g–i): VE_{DIS} is exclusively mediated by a reduction in susceptibility to infection. The columns correspond to assumptions that the single-dose efficacy (SDE) is low (left column, VE_{DIS_1} = 18%), moderate (center column, VE_{DIS_1} = 45%) or high (right column, VE_{DIS_1} = 72%), corresponding 20, 50 or 80% of the 90% efficacy that is assumed following two doses of vaccine, respectively.

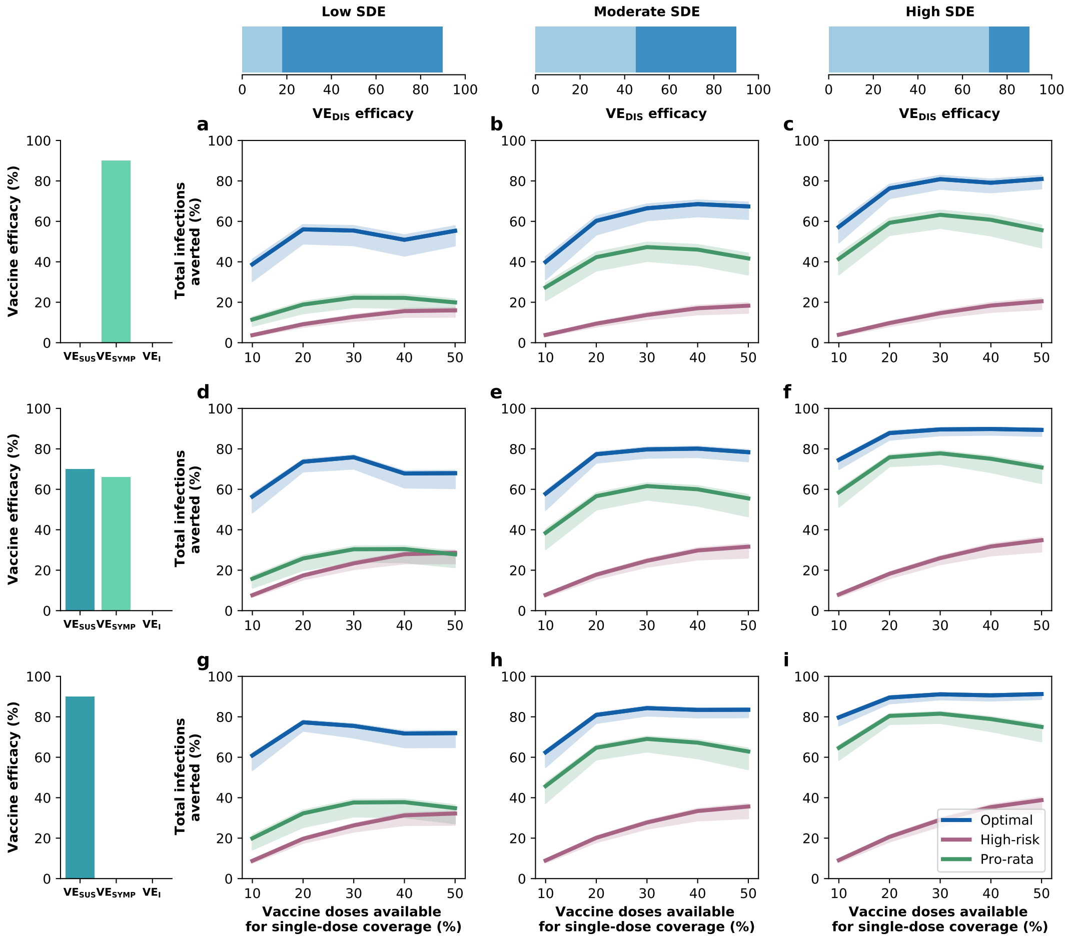

Supplementary Figure 11: Percentage of cumulative infections averted for different vaccine profiles assuming asymptomatic infections are 30% as infectious as symptomatic ones. Percentage of cumulative infections averted for the optimal allocation strategy to minimize infections (blue), the high-risk strategy (pink) and the pro-rata strategy (green) strategies with enough vaccine to cover 10–50% of the population with one dose. Each row represents a different breakdown of vaccine efficacy against disease after two doses $VE_{DIS} = 90\%$ as a function of the vaccine efficacy reducing susceptibility to infection, VE_{SUS}, and the vaccine efficacy reducing the probability of developing COVID-19 symptoms upon infection, VE_{SYMP}. Top row (a–c): VE_{DIS} is exclusively mediated by a reduction in symptoms upon infection. Middle row (d–f): VE_{DIS} is mediated by a combination of reduction in susceptibility to infection and reduction of symptoms upon infection. Bottom row (g–i): VE_{DIS} is exclusively mediated by a reduction in susceptibility to infection. The columns correspond to assumptions that the single-dose efficacy (SDE) is low (left column, $VE_{DIS_1} = 18\%$), moderate (center column, $VE_{DIS_1} = 45\%$) or high (right column, $VE_{DIS_1} = 72\%$), corresponding 20, 50 or 80% of the 90% efficacy that is assumed following two doses of vaccine, respectively. Shaded areas represent central 95% of 1000 simulations (uncertainty intervals, see SM for full details). Here, we assumed an effective reproductive number $R_{eff} = 1.1$.

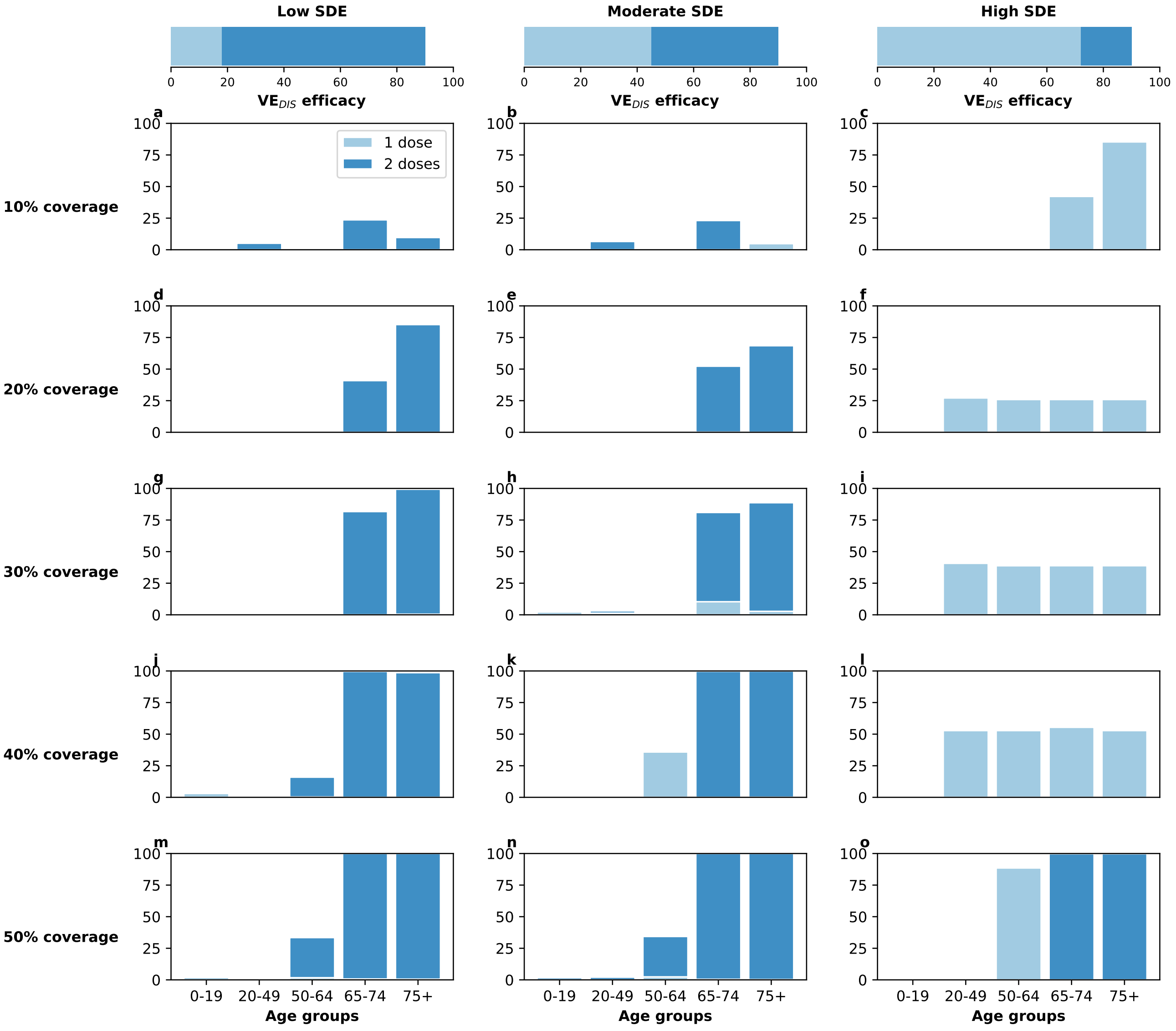

Supplementary Figure 12: Optimal vaccine allocation strategies with different levels of coverage assuming 10% of the population has pre-existing immunity. For each plot, the bars represent the percentage of each age group vaccinated with a single dose (light blue) and two doses (dark blue) when there is enough vaccine to cover 10% to 50% (as indicated by row) of the population with a single dose. The columns correspond to assumptions that the single-dose efficacy (SDE) is low (left column, VE_{DIS_1} = 18%), moderate (center column, VE_{DIS_1} = 45%) or high (right column, VE_{DIS_1} = 72%), corresponding 20, 50 or 80% of the full vaccine efficacy, VE_{DIS} = 90% assumed following two doses of vaccine, respectively. Here, we assumed an effective reproductive number R_{eff} = 1.1.

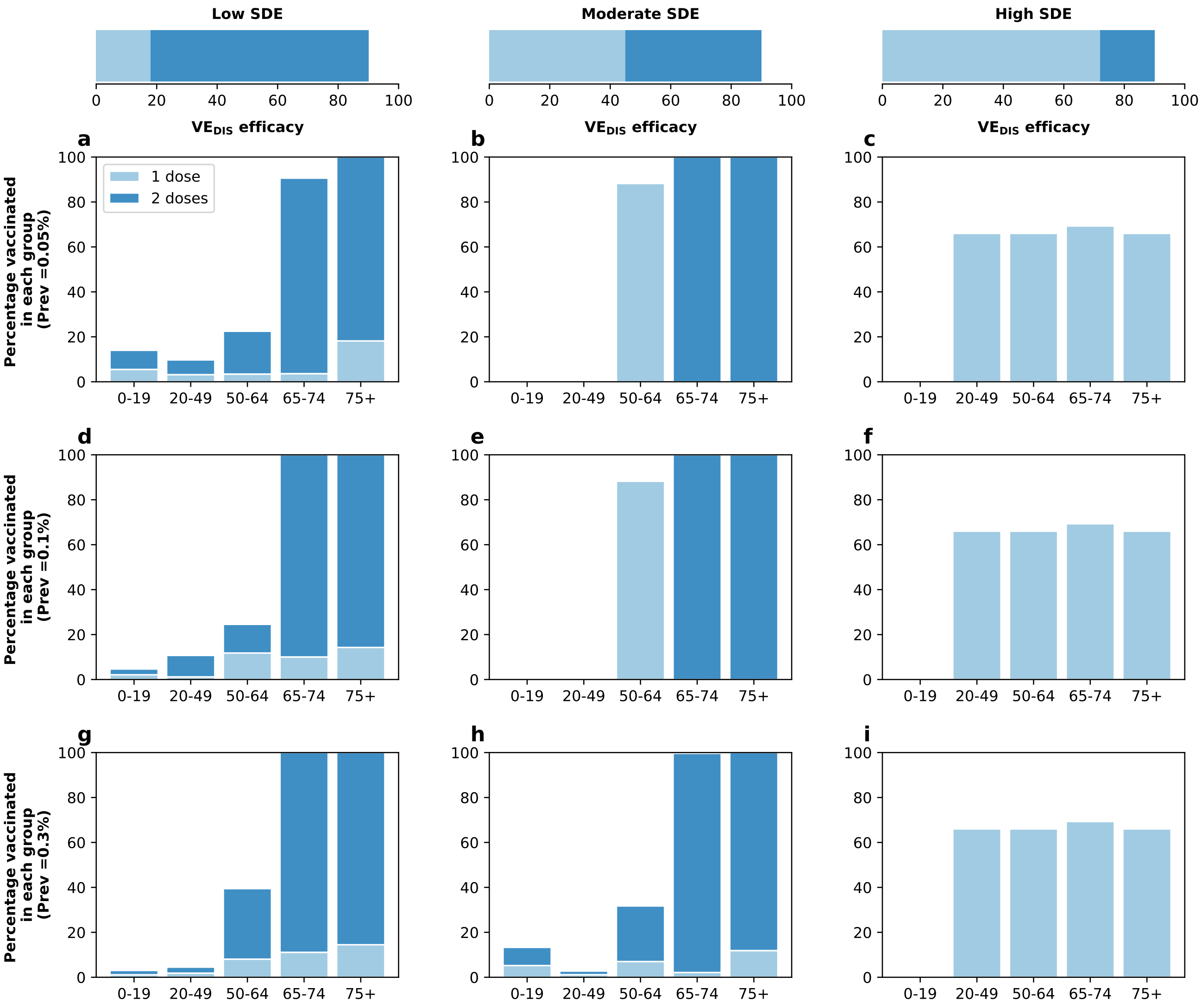

Supplementary Figure 13: Optimal vaccine allocation strategies to minimize deaths for different levels of infection prevalence at the start of vaccination rollout. For each plot, the bars represent the percentage of each age group vaccinated with a single dose (light blue) and two doses (dark blue) when there is enough vaccine to cover 50% of the population with a single dose. Each row represents starting the simulations assuming 0.05% (a–c), 0.1% (d–f) or 0.3% (g–i) of the population is currently infected. The columns correspond to assumptions that the single-dose efficacy (SDE) is low (left column, VE_{DIS_1} = 18%), moderate (center column, VE_{DIS_1} = 45%) or high (right column, VE_{DIS_1} = 72%), corresponding 20, 50 or 80% of the full vaccine efficacy, $VE_{DIS} = 90\%$ assumed following two doses of vaccine, respectively. Here, we assumed an effective reproductive number R_{eff} = 1.1.

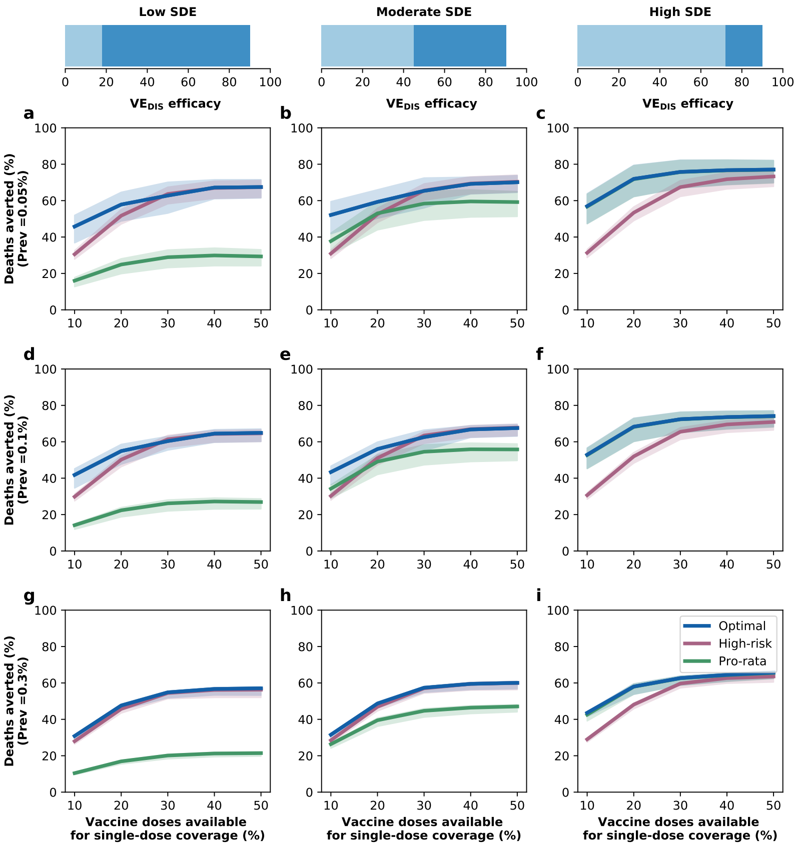

Supplementary Figure 14: Percentage of cumulative deaths averted for different infection prevalence at the start of vaccination rollout. Percentage of cumulative deaths averted for the optimal allocation strategy to minimize deaths (blue), the high-risk strategy (pink) and the pro-rata strategy (green) strategies with enough vaccine to cover 10–50% of the population with one dose. Each row represents starting the simulations assuming 0.05% (a–c), 0.1% (d–f) or 0.3% (g–i) of the population is currently infected. The columns correspond to assumptions that the single-dose efficacy (SDE) is low (left column, VE_{DIS_1} = 18%), moderate (center column, VE_{DIS_1} = 45%) or high (right column, VE_{DIS_1} = 72%), corresponding 20, 50 or 80% of the full vaccine efficacy, $VE_{DIS} = 90\%$ assumed following two doses of vaccine, respectively. Shaded areas represent central 95% of 1000 simulations (uncertainty intervals, see SM for full details). Here, we assumed an effective reproductive number R_{eff} = 1.1.

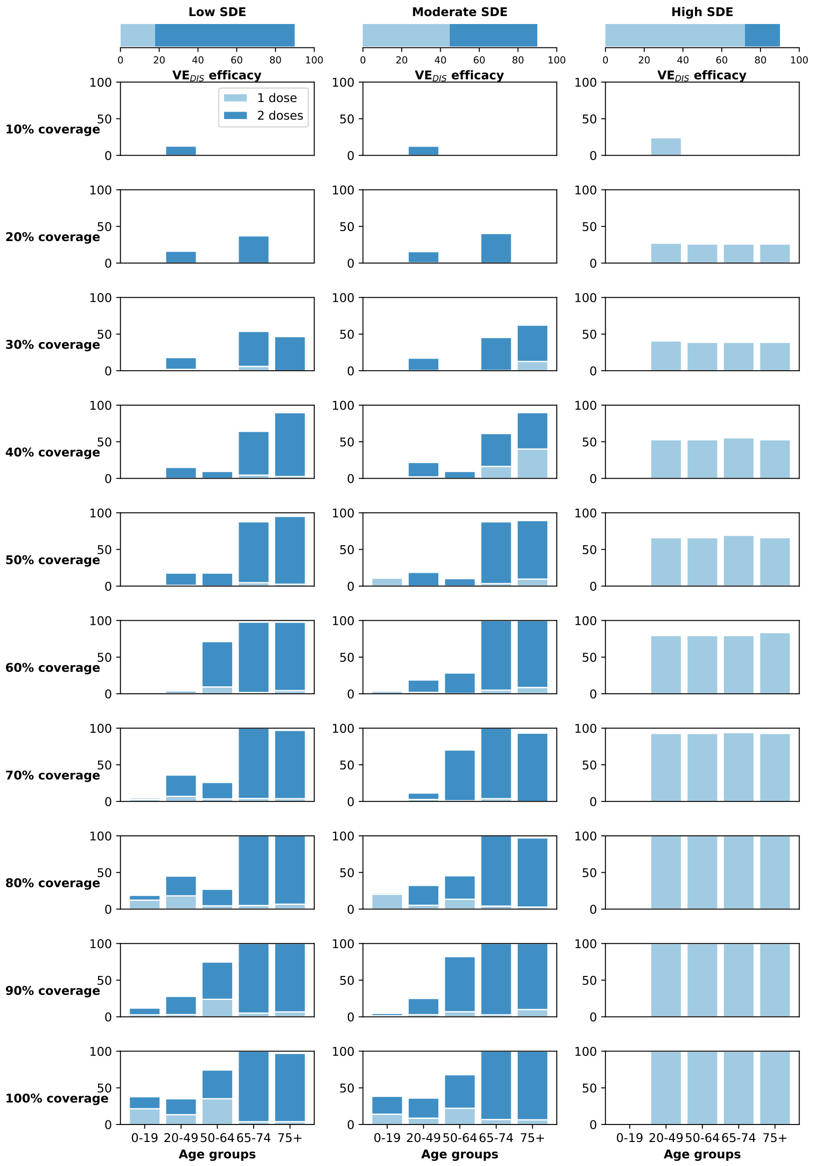

Supplementary Figure 15: Optimal vaccine allocation strategies to minimize deaths with different levels of coverage with vaccination rollout (300K doses per week). For each plot, the bars represent the percentage of each age group vaccinated with a single dose (light blue) and two doses (dark blue) when there is enough vaccine to cover 10% to 100% (as indicated by row) of the population with a single dose. The columns correspond to assumptions that the single-dose efficacy (SDE) is low (left column, VE_{DIS_1} = 18%), moderate (center column, VE_{DIS_1} = 45%) or high (right column, VE_{DIS_1} = 72%), corresponding 20, 50 or 80% of the full vaccine efficacy, VE_{DIS} = 90% assumed following two doses of vaccine, respectively. Here, we assumed an effective reproductive number R_{eff} = 1.1.

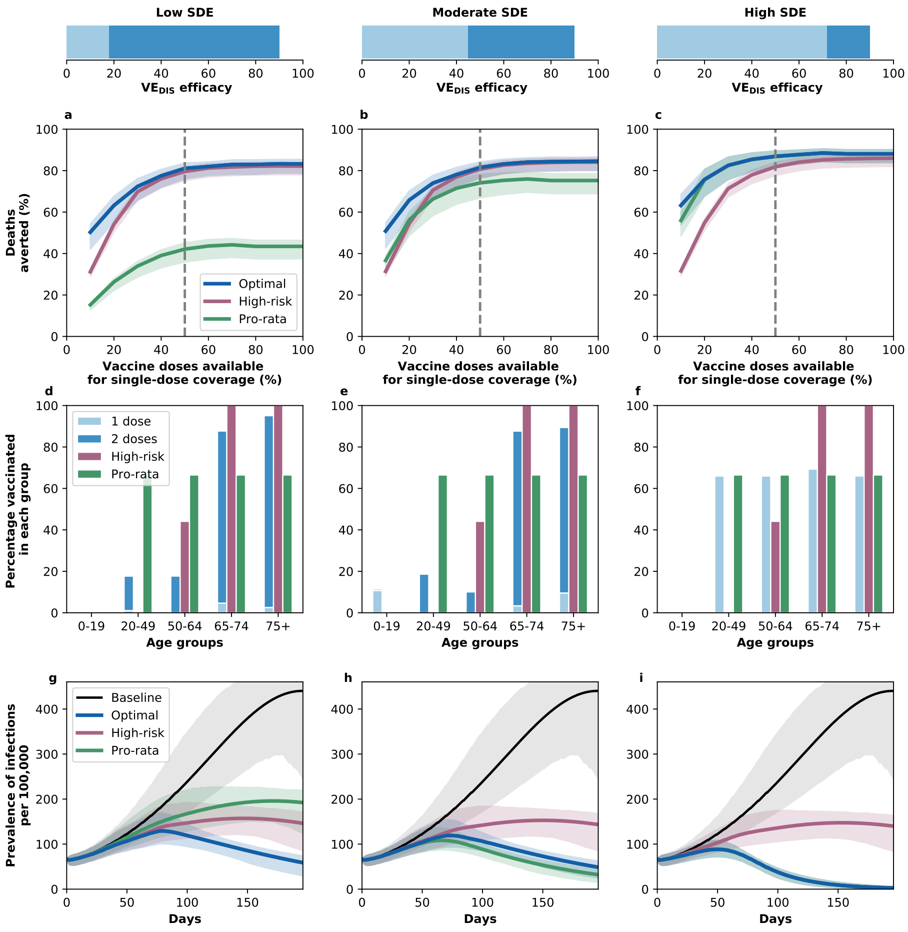

Supplementary Figure 16: Results with a faster vaccination rollout (300K doses per week). a–c. Percentage of deaths averted: Percentage of deaths averted for the optimal allocation strategy to minimize deaths (blue), the high-risk strategy (pink) and the pro-rata strategy (green) strategies with enough vaccine to cover 10–100% of the population with one dose (5–50% with two doses), administering 300K doses per week. At this rate, 100% of the population can be vaccinated with a single dose in our time horizon. d–f. Allocation strategies: Optimal (light and dark blue), high-risk (pink) and pro-rata (green) allocation strategies with enough vaccine to cover 50% of the population with a single dose (25% with two doses). Within each panel, the bars represent the percentage vaccinated in each vaccination group. g–i. Prevalence of infections: Prevalence of active infections (per 100,000) in absence of vaccine (black), with the optimal allocation strategy to minimize deaths (blue), the high-risk strategy (pink) or the pro-rata strategy (green) with enough vaccine to cover 20% of the population with one dose (10% with two doses). Shaded areas represent central 95% of 1000 simulations (uncertainty intervals, see SM for full details). The columns correspond to assumptions that the single-dose efficacy (SDE) is low (left column, $VE_{DIS_1} = 18\%$), moderate (center column, $VE_{DIS_1} = 45\%$) or high (right column, $VE_{DIS_1} = 72\%$), corresponding 20, 50 or 80% of the full vaccine efficacy, $VE_{DIS} = 90\%$ assumed following two doses of vaccine, respectively. Here, we assumed an effective reproductive number $R_{eff} = 1.1$.

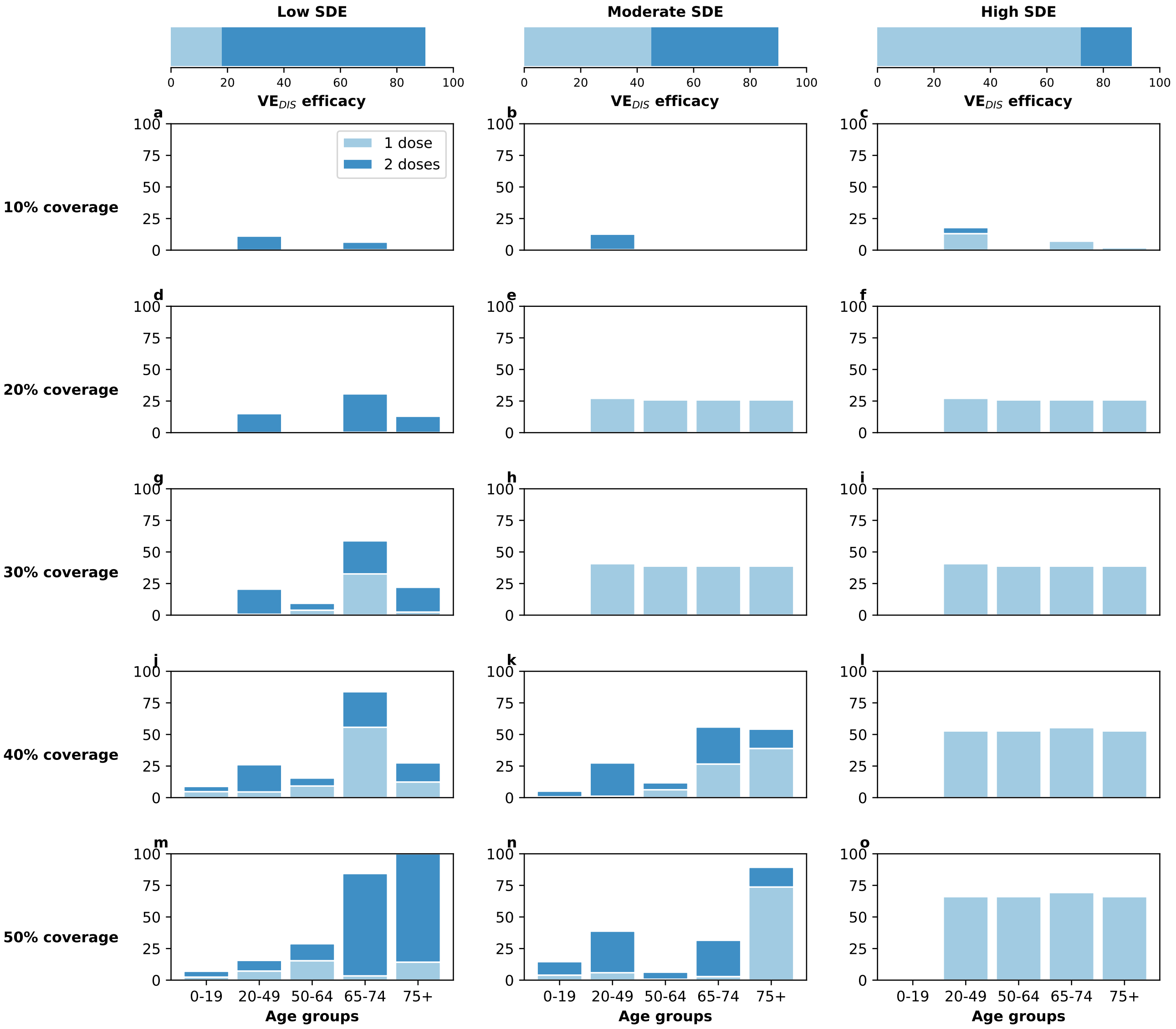

Supplementary Figure 17: Optimal vaccine allocation strategies to minimize deaths with different levels of coverage with a vaccine reducing also infectiousness (VE_I = 70%). For each plot, the bars represent the percentage of each age group vaccinated with a single dose (light blue) and two doses (dark blue) when there is enough vaccine to cover 10% (row A) to 50% (row E) of the population with a single dose. Here, we assumed that the vaccine reduces susceptibility to infection, symptoms given infection and infectiousness with $VE_I = 70\%$. The columns correspond to assumptions that the single-dose efficacy (SDE) is low (left column, VE_{DIS_1} = 18%), moderate (center column, VE_{DIS_1} = 45%) or high (right column, VE_{DIS_1} = 72%), corresponding 20, 50 or 80% of the full vaccine efficacy, $VE_{DIS} = 90\%$ assumed following two doses of vaccine, respectively.

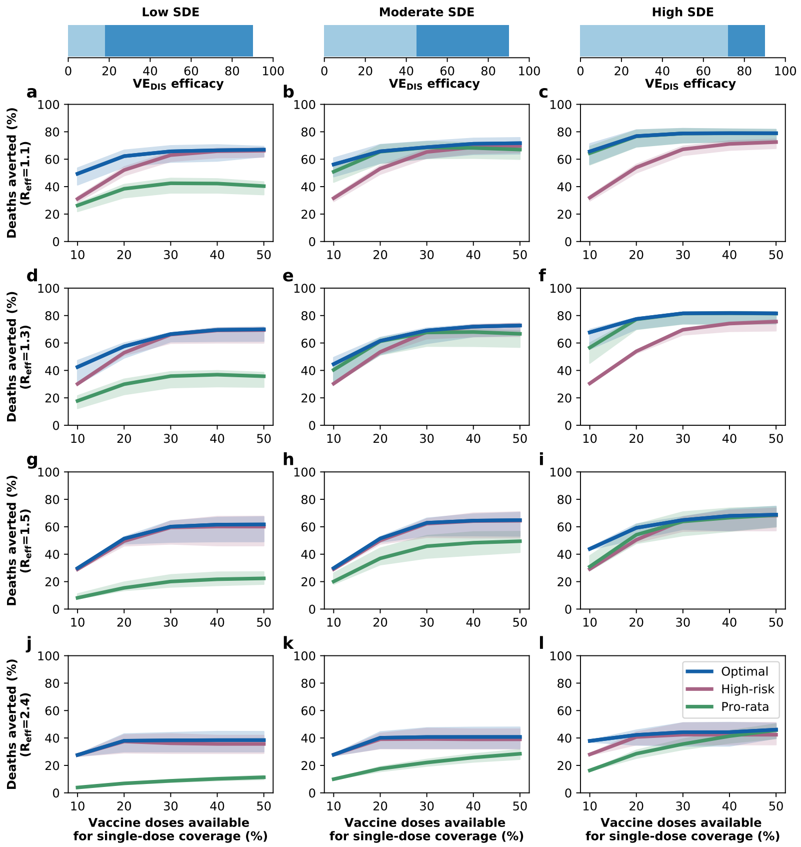

Supplementary Figure 18: Percentage of deaths averted for different levels of SARS-CoV-2 transmission with a vaccine reducing also infectiousness. Percentage of deaths averted for the optimal allocation strategy to minimize deaths (blue), the high-risk strategy (pink) and the pro-rata strategy (green) strategies with enough vaccine to cover 10-50% of the population with one dose. Each row represents a different level of SARS-CoV-2 transmission resulting in an effective reproductive number $R_{eff} = 1.1$ (a–c), 1.3 (d–f), 1.5 (g–i) or 2.4 (j–l). Here, we assumed that the vaccine reduces susceptibility to infection, symptoms given infection and infectiousness with $VE_I = 70\%$.The columns correspond to assumptions that the single-dose efficacy (SDE) is low (left column, $VE_{DIS_1} = 18\%$), moderate (center column, $VE_{DIS_1} = 45\%$) or high (right column, $VE_{DIS_1} = 72\%$), corresponding 20, 50 or 80% of the full vaccine efficacy, $VE_{DIS} = 90\%$ assumed following two doses of vaccine, respectively. Shaded areas represent central 95% of 1000 simulations (uncertainty intervals, see SM for full details).

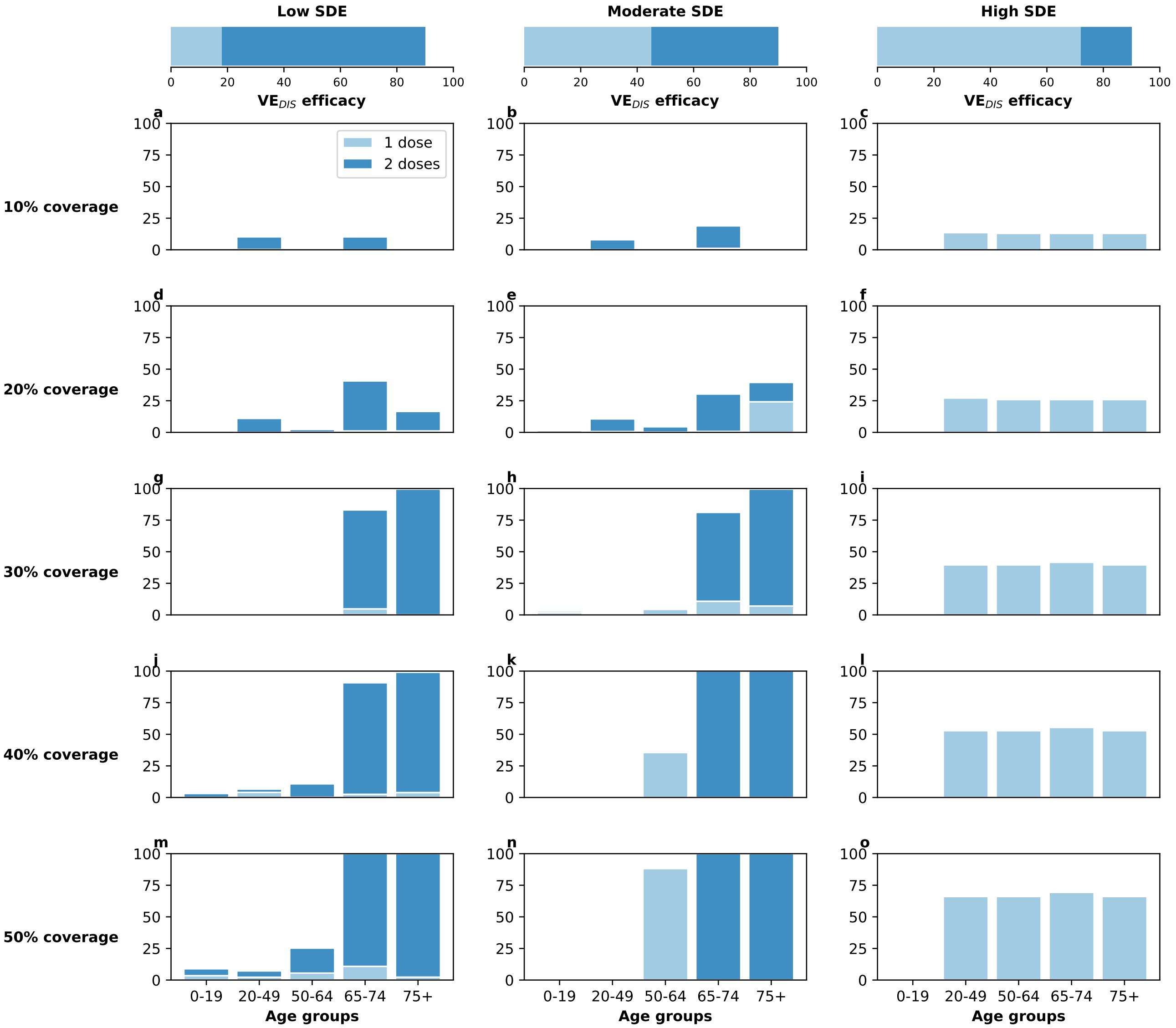

Supplementary Figure 19: Optimal vaccine allocation strategies to minimize deaths with different levels of coverage assuming a different distribution of pre-existing immunity, similar to the distribution of cases observed in WA state as of February 2021 [2]. For each plot, the bars represent the percentage of each age group vaccinated with a single dose (light blue) and two doses (dark blue) when there is enough vaccine to cover 10% (row A) to 50% (row E) of the population with a single dose. The columns correspond to assumptions that the single-dose efficacy (SDE) is low (left column, VE_{DIS_1} = 18%), moderate (center column, VE_{DIS_1} = 45%) or high (right column, VE_{DIS_1} = 72%), corresponding 20, 50 or 80% of the full vaccine efficacy, $VE_{DIS} = 90\%$ assumed following two doses of vaccine, respectively.

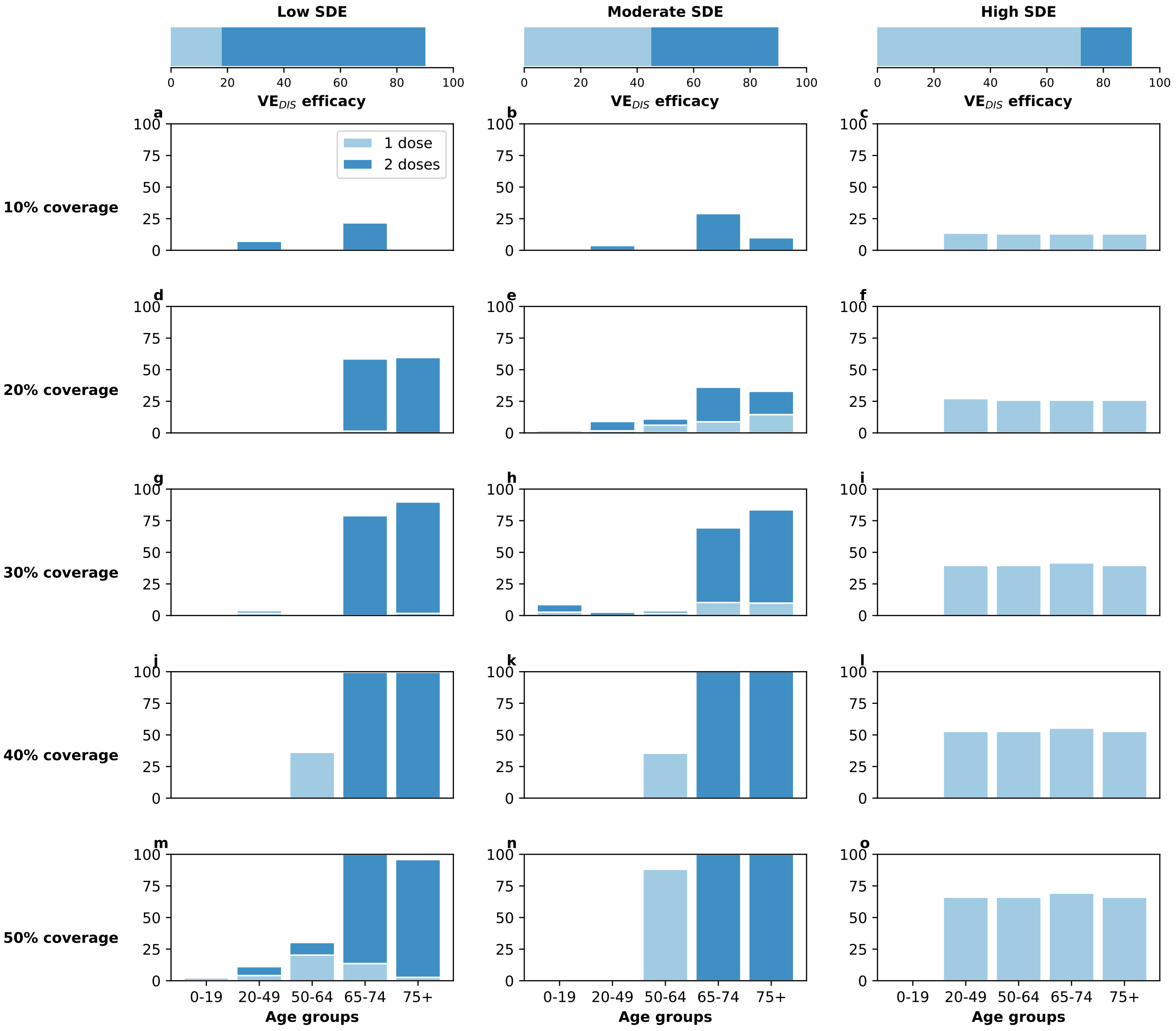

Supplementary Figure 20: Optimal vaccine allocation strategies to minimize deaths with different levels of coverage assuming a different distribution of pre-existing immunity, similar to the distribution of cases observed in two Indian States as of July 2020 [3]. For each plot, the bars represent the percentage of each age group vaccinated with a single dose (light blue) and two doses (dark blue) when there is enough vaccine to cover 10% (row A) to 50% (row E) of the population with a single dose. Here, we assumed that the vaccine reduces susceptibility to infection, symptoms given infection and infectiousness with $VE_I = 70\%$. The columns correspond to assumptions that the single-dose efficacy (SDE) is low (left column, VE_{DIS_1} = 18%), moderate (center column, VE_{DIS_1} = 45%) or high (right column, VE_{DIS_1} = 72%), corresponding 20, 50 or 80% of the full vaccine efficacy, $VE_{DIS} = 90\%$ assumed following two doses of vaccine, respectively.

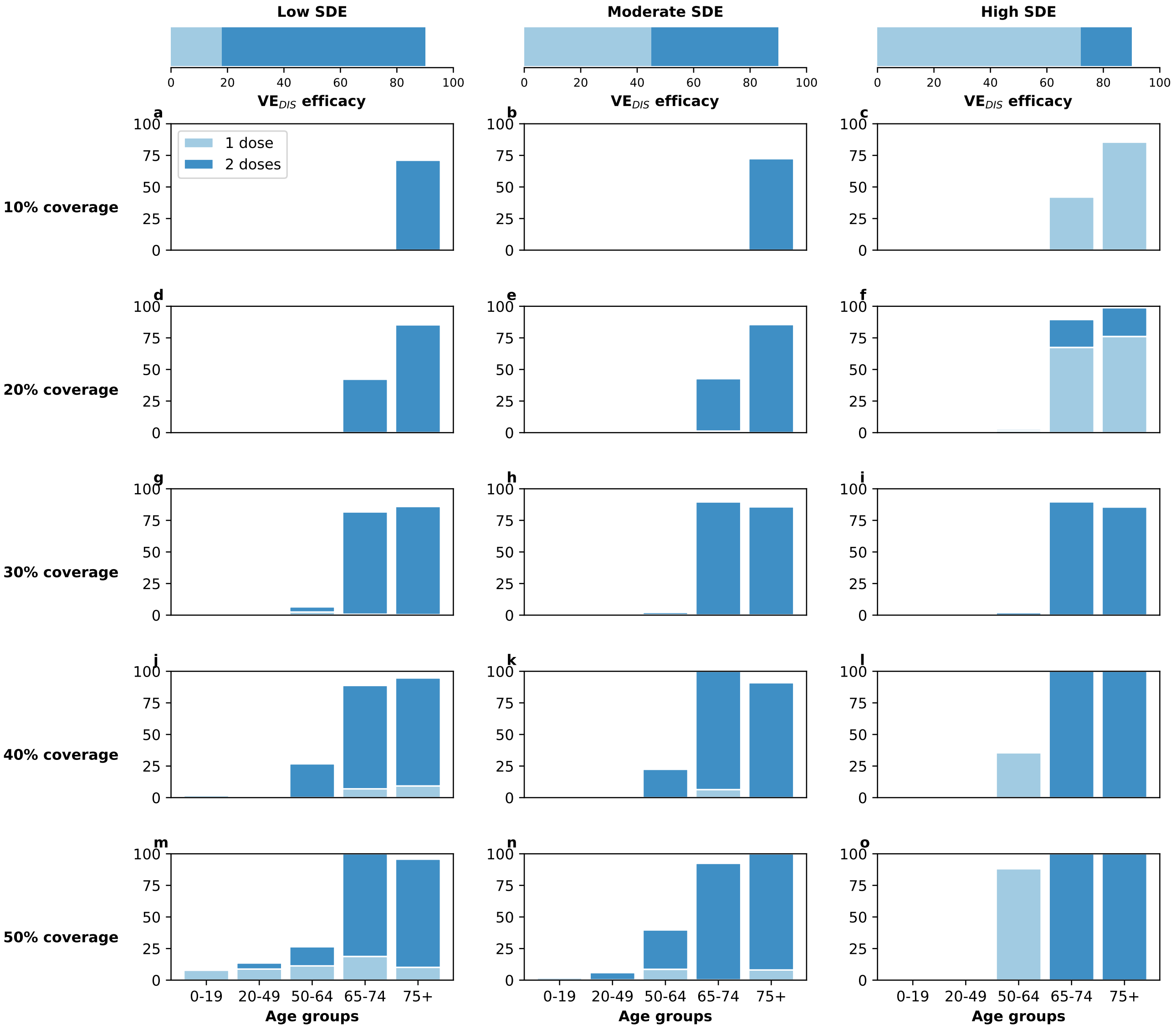

Supplementary Figure 21: Optimal vaccine allocation strategies to minimize deaths assuming increased baseline transmission. For each plot, the bars represent the percentage of each age group vaccinated with a single dose (light blue) and two doses (dark blue) when there is enough vaccine to cover 10% (row A) to 50% (row E) of the population with a single dose. The columns correspond to assumptions that the single-dose efficacy (SDE) is low (left column, VE_{DIS_1} = 18%), moderate (center column, VE_{DIS_1} = 45%) or high (right column, VE_{DIS_1} = 72%), corresponding 20, 50 or 80% of the full vaccine efficacy, $VE_{DIS} = 90\%$ assumed following two doses of vaccine, respectively. Here, we assumed an increased baseline transmission with a basic reproductive number $R_0 = 4$ and social distancing interventions resulting in an effective reproductive number $R_{eff} = 1.5$.

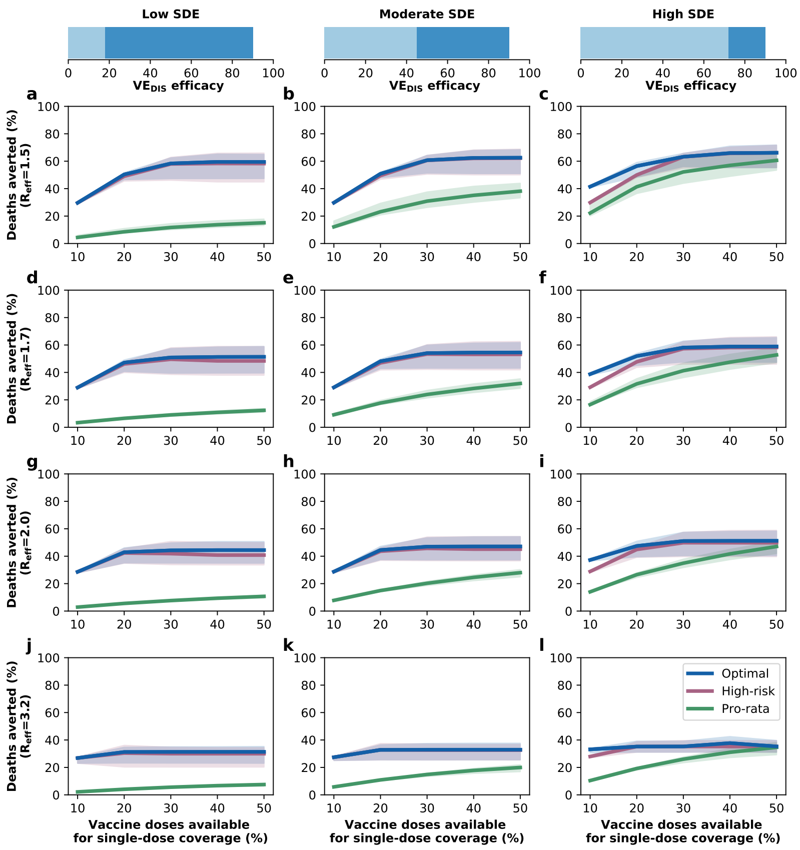

Supplementary Figure 22: Percentage of deaths averted for different levels of SARS-CoV-2 transmission assuming increased baseline transmission. Percentage of deaths averted for the optimal allocation strategy to minimize deaths (blue), the high-risk strategy (pink) and the pro-rata strategy (green) strategies with enough vaccine to cover 10-50% of the population with one dose. Here, we assumed that in absence of any social distancing intervention, the basic reproductive number $R_0 = 4$. Each row represents a different level of social distancing interventions resulting in an effective reproductive number R_{eff} = 1.5 (a–c), 1.7 (d–f), 2.0 (g–i) or 3.2 (j–l). The columns correspond to assumptions that the single-dose efficacy (SDE) is low (left column, VE_{DIS_1} = 18%), moderate (center column, VE_{DIS_1} = 45%) or high (right column, VE_{DIS_1} = 72%), corresponding 20, 50 or 80% of the full vaccine efficacy, $VE_{DIS} = 90\%$ assumed following two doses of vaccine, respectively. Shaded areas represent central 95% of 1000 simulations (uncertainty intervals, see SM for full details).

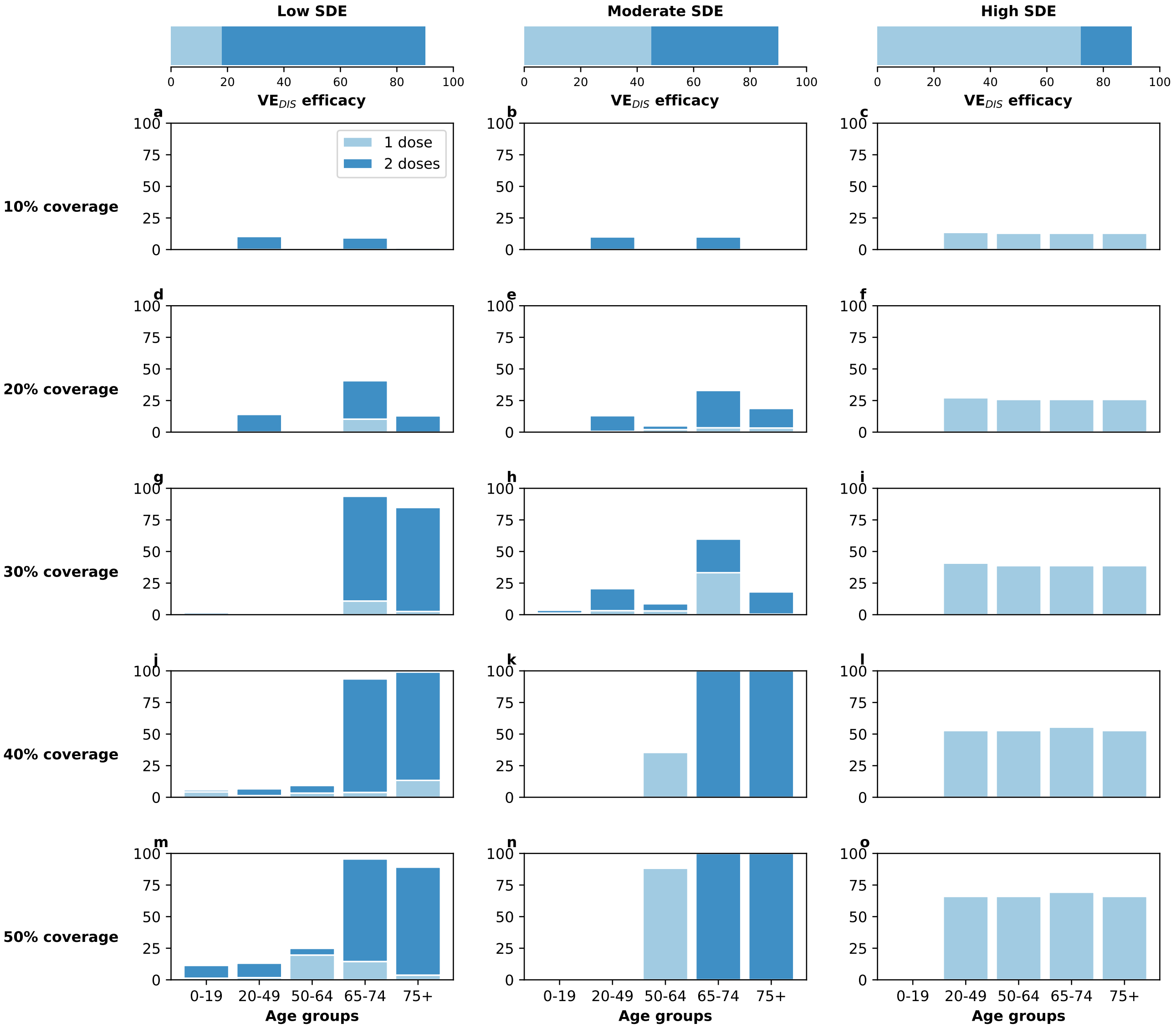

Supplementary Figure 23: Optimal vaccine allocation strategies to minimize deaths assuming a lower vaccine efficacy, $VE_{DIS} = 72\%$, after two doses. For each plot, the bars represent the percentage of each age group vaccinated with a single dose (light blue) and two doses (dark blue) when there is enough vaccine to cover 10% (row A) to 50% (row E) of the population with a single dose. The columns correspond to assumptions that the single-dose efficacy (SDE) is low (left column, $VE_{DIS_1} = 14\%$), moderate (center column, $VE_{DIS_1} = 35\%$) or high (right column, $VE_{DIS_1} = 58\%$), corresponding 20, 50 or 80% of the full vaccine efficacy $VE_{DIS} = 72\%$ assumed following two doses of vaccine, respectively.

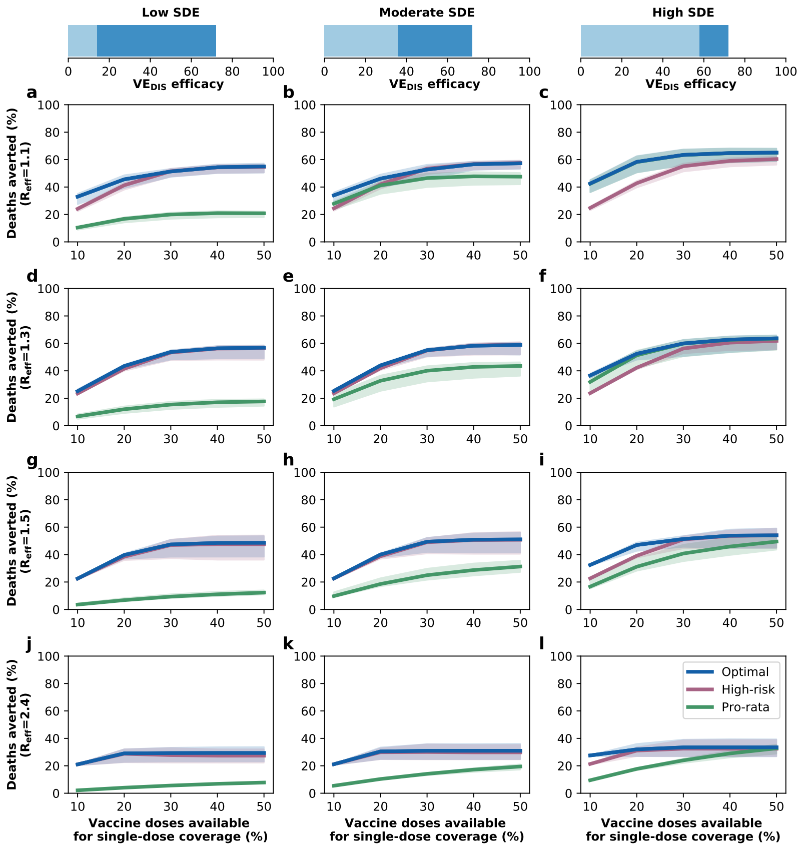

Supplementary Figure 24: Percentage of deaths averted for different levels of SARS-CoV-2 transmission assuming vaccine efficacy against disease after two doses $VE_{DIS} = 72\%$. Percentage of deaths averted for the optimal allocation strategy to minimize deaths (blue), the high-risk strategy (pink) and the pro-rata strategy (green) strategies with enough vaccine to cover 10-50% of the population with one dose. Each row represents a different level of social distancing interventions resulting in R_{eff} = 1.5 (a–c), 1.7 (d–f), 2.0 (g–i) or 3.2 (j–l). The columns correspond to assumptions that the single-dose efficacy (SDE) is low (left column, VE_{DIS_1} = 14%), moderate (center column, VE_{DIS_1} = 35%) or high (right column, VE_{DIS_1} = 58%), corresponding 20, 50 or 80% of the full vaccine efficacy $VE_{DIS} = 72\%$ assumed following two doses of vaccine, respectively. Shaded areas represent central 95% of 1000 simulations (uncertainty intervals, see SM for full details).

Supplemental Tables

	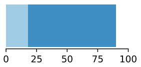		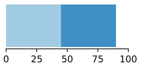		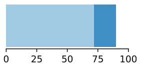		TOTAL	
	$VE_{DIS_1} = 18\%$		$VE_{DIS_1} = 45\%$		$VE_{DIS_1} = 72\%$		$VE_{DIS} = 90\%$	
	VE_{SUS_1}	VE_{SYMP_1}	VE_{SUS_1}	VE_{SYMP_1}	VE_{SUS_1}	VE_{SYMP_1}	VE_{SUS}	VE_{SYMP}
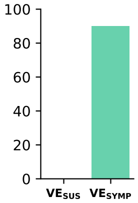 Vaccine mediated mainly by reducing symptoms upon infection	0%	18%	0%	45%	0%	72%	0%	90%
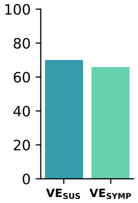 Vaccine mediated by reducing symptoms upon infection and reducing susceptibility to infection	9%	10%	26%	26%	50%	44%	70%	66%
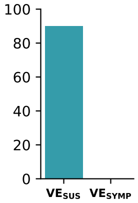 Vaccine mediated mainly by reducing susceptibility to infection	18%	0%	45%	0%	72%	0%	90%	0%

Supplementary Table 1: Description of vaccine efficacies used in the model. The vaccine efficacies against disease after one and two doses are denoted VE_{DIS_1} and VE_{DIS} respectively. Vaccine efficacies reducing susceptibility to infection after one and two doses are denoted VE_{SUS_1} and VE_{SUS} respectively. Vaccine efficacies reducing COVID-19 symptoms upon infection after one and two doses are denoted VE_{SYMP_1} and VE_{SYMP} respectively.

Parameter	Meaning	Value (Range)	Reference
N	Total population	7.615 M	[20]
-	Age distribution of the population	-	[23]
-	Total number of general hospital beds	12906	[30]
-	Total number of ICU hospital beds	1390	[30]
$1/\gamma_E$	mean duration of latent period	3 (1.5–4.5) d	[4, 5]
$1/\gamma_P$	mean pre-symptomatic period	2 (1–3) d	[6]
$1/\gamma_A$	mean infectious period of asymptomatic infections	6 (3–8) d	assumed[a]
$1/\gamma_I$	mean infectious period of symptomatic infections not requiring hospitalization after developing symptoms	4 (2–5) d	[7, 8]
$1/\gamma_H$	mean duration of non-ICU hospitalization	age-stratified	[9]
$1/\gamma_C$	mean duration of ICU hospitalization	age-stratified	[9]
k_{0-14}	proportion of infections that are symptomatic	0.25 (0.4–0.8)	[10]
k_{15+}	proportion of infections that are symptomatic	0.60 (0.4–0.8)	[9, 11, 12]
h	proportion of symptomatic infections requiring hospitalization	age-stratified	[13]
c	proportion of hospitalizations requiring ICU	age-stratified	[13]
d	proportion of all hospitalizations resulting in death	age-stratified	[14]
r_A	relative infectiousness of asymptomatic infections [b]	0.75 (0.3)	[9]
r_H	relative infectiousness of hospitalized infections	0	assumed
r_P	relative infectiousness of pre-symptomatic infections[c]	1 (0.7–1.3)	[15]
m_{0-14}	relative susceptibility for those aged 0–14	0.56	[10, 5]
m_{15-64}	relative susceptibility for those aged 15–64	1	[10, 5]
m_{65+}	relative susceptibility for those age 65+	2.7	[16]
σ	mean time from symptom onset to hospitalization	3.8 d	[17]
R_0	basic reproductive number	3	[18, 19]
R_{eff}	effective reproductive number at the beginning of vaccination	1.1, 1.3, 1.5, 2.4	assumed[d]
β	transmission coefficient	calculated	–
$\mathcal{M}$	contact matrix	–	[1]
N	total population	7,615,000	[20]
$R(0)$	proportion of the population immune at $t = 0$	0.2 (0.1)	[21]
$I(0)$	infected proportion of the total population at $t = 0$	0.001 (0.0005 and 0.003)	[2]

[a] assumed to match the duration of infectiousness of symptomatic infections
[b] with respect to symptomatic not hospitalized infections
[c] with respect to symptomatic not hospitalized infections
[d] see table 3 for details.

Supplementary Table 2: Description of parameters used in the model.

R_{eff}	Home	Work	Other locations	School
1.1	1	0.6	0.2	0.1
1.3	1	0.6	0.4	0.1
1.5	1	0.6	0.5	0.5
2.4	1	1	1	1

Supplementary Table 3: Multipliers used based on the contact matrices given in Prem et al. [1].

Supplementary references

[1] Prem, K., Cook, A. R. & Jit, M. Projecting social contact matrices in 152 countries using contact surveys and demographic data. *PLoS Computational Biology* **13**, e1005697 (2017).

[2] Washington State Department of Health. COVID-19 Data Dashboard (2021). URL `https://www.doh.wa.gov/Emergencies/COVID19/DataDashboard{\#}dashboard`.

[3] Laxminarayan, R. *et al.* Epidemiology and transmission dynamics of covid-19 in two indian states. *Science* **370**, 691–697 (2020). URL `https://science.sciencemag.org/content/370/6517/691`. `https://science.sciencemag.org/content/370/6517/691.full.pdf`.

[4] Lauer, S. A. *et al.* The incubation period of coronavirus disease 2019 (COVID-19) from publicly reported confirmed cases: Estimation and application. *Annals of Internal Medicine* (2020). URL `http://www.ncbi.nlm.nih.gov/pubmed/32150748`.

[5] Zhang, J. *et al.* Changes in contact patterns shape the dynamics of the COVID-19 outbreak in China. *Science* **368**, 1481–1486 (2020).

[6] Wei, W. E. *et al.* Presymptomatic Transmission of SARS-CoV-2-Singapore. *Morbidity and Mortality Weekly Report* **69**, 411–415 (2020).

[7] Du, Z. *et al.* The serial interval of COVID-19 from publicly reported confirmed cases. *Emerging Infectious Diseases* **26**, 1341–1343 (2020).

[8] Bi, Q. *et al.* Epidemiology and transmission of COVID-19 in 391 cases and 1286 of their close contacts in Shenzhen, China: a retrospective cohort study. *Lancet Infectious Diseases* (2020).

[9] CDC. COVID-19 Pandemic Planning Scenarios. URL `https://www.cdc.gov/coronavirus/2019-ncov/hcp/planning-scenarios.html`.

[10] Viner, R. M. *et al.* Susceptibility to SARS-CoV-2 infection among children and adolescents compared with adults: A systematic review and meta-analysis. *JAMA Pediatrics* **175**, 143–156 (2021).

[11] Lee, Y.-H., Hong, C. M., Kim, D. H., Lee, T. H. & Lee, J. Clinical course of asymptomatic and mildly symptomatic patients with coronavirus disease admitted to community treatment centers, South Korea. *Emerging Infectious Diseases* **Oct** (2020). URL `https://wwwnc.cdc.gov/eid/article/26/10/20-1620{\_}article`.

[12] Mizumoto, K., Kagaya, K., Zarebski, A. & Chowell, G. Estimating the asymptomatic proportion of coronavirus disease 2019 (COVID-19) cases on board the Diamond Princess cruise ship, Yokohama, Japan, 2020. *Euro Surveillance* **25** (2020). URL doi.org/10.2807/1560-7917.

[13] Ferguson, N. M. *et al.* Impact of non-pharmaceutical interventions (NPIs) to reduce COVID-19 mortality and healthcare demand (2020). URL https://doi.org/10.25561/77482.

[14] Salje, H. *et al.* Estimating the burden of sars-cov-2 in france. *Science* **369**, 208–211 (2020). URL https://science.sciencemag.org/content/369/6500/208. https://science.sciencemag.org/content/369/6500/208.full.pdf.

[15] Slifka, M. K. & Gao, L. Is presymptomatic spread a major contributor to COVID-19 transmission? *Nature Medicine* **26**, 1531–1533 (2020). URL http://dx.doi.org/10.1038/s41591-020-1046-6.

[16] Bi, Q. *et al.* Household transmission of SARS-CoV-2: Insights from a population-based serological survey. *medRxiv* (2021). URL https://www.medrxiv.org/content/10.1101/2020.11.04.20225573v2.

[17] Zhang, J. *et al.* Evolving epidemiology and transmission dynamics of coronavirus disease 2019 outside hubei province, china: a descriptive and modelling study. *The Lancet Infectious Diseases* **20**, 793–802 (2020). URL https://doi.org/10.1016/S1473-3099(20)30230-9.

[18] Wu, J. T., Leung, K. & Leung, G. M. Nowcasting and forecasting the potential domestic and international spread of the 2019-nCoV outbreak originating in Wuhan, China: a modelling study. *Lancet* **395**, 689–697 (2020). URL http://dx.doi.org/10.1016/S0140-6736(20)30260-9.

[19] Zhao, S. *et al.* Preliminary estimation of the basic reproduction number of novel coronavirus (2019-nCoV) in China, from 2019 to 2020: A data-driven analysis in the early phase of the outbreak. *International Journal of Infectious Diseases* **92**, 214–217 (2020). URL https://doi.org/10.1016/j.ijid.2020.01.050.

[20] United States Census Bureau. Washington (2020). URL https://data.census.gov/cedsci/profile?q=Washington{\&}g=0400000US53.

[21] Centers for Disease Control and Prevention. Estimated disease burden of COVID-19 (2021). URL https://www.cdc.gov/coronavirus/2019-ncov/cases-updates/burden.html.

[22] Matrajt, L., Eaton, J., Leung, T. & Brown, E. R. Vaccine optimization for COVID-19: Who to vaccinate first? *Science Advances* **7**, eabf1374 (2021). URL http://advances.sciencemag.org/content/7/6/eabf1374.

[23] Population Pyramid. Population of United States of America 2020 (2020). URL https://www.populationpyramid.net/united-states-of-america/2020/.

[24] Inslee Washington Governor Jay. Proclamation by the governor: Amending proclamations 20-05 (2020). URL https://www.governor.wa.gov/sites/default/files/proclamations/20-25CoronovirusStaySafe-StayHealthy{\%}28tmp{\%}29{\%}28002{\%}29.pdf.

[25] Washington Governor Jay Inslee. Inslee announces statewide shutdown of restaurants, bars and expanded social gathering limits (2020). URL https://medium.com/wagovernor/inslee-announces-statewide-shutdown-of-restaurants-bars-and-expanded-s

[26] Inslee, W. G. J. Inslee statement on statewide shutdown of restaurants, bars and limits on size of gatherings expanded (2020). URL https://www.governor.wa.gov/news-media/inslee-statement-statewide-shutdown-restaurants-bars-and-limits-size-g

[27] Jay, I. W. G. Proclamation by the governor: Amending proclamations 20-05 and 20-06 (2020). URL https://www.governor.wa.gov/sites/default/files/20-07Coronavirus{\%}28tmp{\%}29.pdf.

[28] WA Govenor's Office. Inslee announces school closures in King, Snohomish and Pierce counties (2020). URL https://medium.com/wagovernor/inslee-announces-school-closures-in-king-snohomish-and-pierce-counties-

[29] Pan, Y. *et al.* Quantifying human mobility behaviour changes during the COVID-19 outbreak in the United States. *Scientific Reports* **10**, 1–9 (2020). URL https://doi.org/10.1038/s41598-020-77751-2.

[30] Washington State Coronavirus Response. COVID-19 risk assessment dashboard (2020). URL https://coronavirus.wa.gov/what-you-need-know/covid-19-risk-assessment-dashboard.

[31] State of California. COVID-19 data and tools (2020). URL https://covid19.ca.gov/data-and-tools/.

[32] Halloran, M. E., Struchiner, C. J. & Longini, I. M. Study designs for evaluating different efficacy and effectiveness aspects of vaccines. *American Journal of Epidemiology* **146**, 789–803 (1997).

[33] Mehrotra, D. V. *et al.* Clinical endpoints for evaluating efficacy in covid-19 vaccine trials. *Annals of Internal Medicine* (2020). URL https://doi.org/10.7326/M20-6169.

[34] Audet, C. & Hare, W. *Derivative-Free and Blackbox Optimization* (Springer, Cham, Switzerland, 2017).

[35] The Sage Developers. SageMath, the Sage Mathematics Software System (Version 9.1) (2020). URL https://www.sagemath.org.

[36] Kotz, S., Balakrishnan, N. & Johnson, N. L. *Continuous Multivariate Distributions. Volume 1: Models and Applications* (Wiley, 2000).

[37] Gao, F. & Han, L. Implementing the Nelder-Mead simplex algorithm with adaptive parameters. *Computational Optimization and Applications* **51**, 259–277 (2012).

[38] Virtanen, P. *et al.* SciPy 1.0: Fundamental Algorithms for Scientific Computing in Python. *Nature Methods* **17**, 261–272 (2020). URL https://doi.org/10.1038/s41592-019-0686-2.
